# A gene expression profile of stem cell pluripotentiality and differentiation is conserved across diverse solid and hematopoietic cancers

**DOI:** 10.1186/gb-2012-13-8-r71

**Published:** 2012-08-21

**Authors:** Nathan P Palmer, Patrick R Schmid, Bonnie Berger, Isaac S Kohane

**Affiliations:** 1 Computer Science and Artificial Intelligence Laboratory, Massachusetts Institute of Technology, 77 Massachusetts Avenue, Cambridge MA 02139, USA; 2 Department of Mathematics, Massachusetts Institute of Technology, 77 Massachusetts Avenue, Cambridge MA 02139, USA; 3 Center for Biomedical Informatics, Harvard Medical School, 25 Shattuck Street, Boston MA 02115, USA

## Abstract

**Background:**

Understanding the fundamental mechanisms of tumorigenesis remains one of the most pressing problems in modern biology. To this end, stem-like cells with tumor-initiating potential have become a central focus in cancer research. While the cancer stem cell hypothesis presents a compelling model of self-renewal and partial differentiation, the relationship between tumor cells and normal stem cells remains unclear.

**Results:**

We identify, in an unbiased fashion, mRNA transcription patterns associated with pluripotent stem cells. Using this profile, we derive a quantitative measure of stem cell-like gene expression activity. We show how this 189 gene signature stratifies a variety of stem cell, malignant and normal tissue samples by their relative plasticity and state of differentiation within Concordia, a diverse gene expression database consisting of 3,209 Affymetrix HGU133+ 2.0 microarray assays. Further, the orthologous murine signature correctly orders a time course of differentiating embryonic mouse stem cells. Finally, we demonstrate how this stem-like signature serves as a proxy for tumor grade in a variety of solid tumors, including brain, breast, lung and colon.

**Conclusions:**

This core stemness gene expression signature represents a quantitative measure of stem cell-associated transcriptional activity. Broadly, the intensity of this signature correlates to the relative level of plasticity and differentiation across all of the human tissues analyzed. The fact that the intensity of this signature is also capable of differentiating histological grade for a variety of human malignancies suggests potential therapeutic and diagnostic implications.

## Background

There have been numerous investigations into the relationship between normal organogenesis programs and malignancy, particularly with respect to the stem cell properties of self-renewal and pluripotentiality [1-3]. At the molecular level, certain malignant tumors and developing tissues have been shown to exhibit shared transcription factor activity, regulation of chromatin structure, signaling characteristics and gene expression characteristics [4]. Likewise, enrichment patterns of well-characterized gene sets have been observed to be similar in stem cells and breast cancers, bladder cancers and poorly differentiated glioblastomas [5]. In addition, a variety of stem cell populations have been identified that are specific to individual tissues, yet share some of the same gene expression characteristics of embryonic stem (ES) cells [6]. However, multiple controversies continue to circulate around the role of particular genes in stem cells versus differentiated tissues (for example, N-cadherin [7]), and the extent to which the activation of various stem cell-like programs and pathways occurs across various tissues and diseases.

The cancer stem cell hypothesis asserts a model of tumorigenesis that may tie some of these observations together [8]. By implying a hierarchical organization of tumor growth that closely reflects normal tissue development, the hypothesis simultaneously accounts for the high degree of functional heterogeneity observed in solid tumors [9,10], as well as the fact that only a small fraction of malignant cells retain tumor-initiating potential [8]. Under these assumptions, expression profiles derived from resected tumor samples (comprising both the cancer stem cells and their differentiated progeny) should broadly resemble those of the normal tissue of origin, with a degree of stem cell like activity also apparent.

Originally identified in hematopoietic cancers, leukemic stem cells were observed to express several markers (CD34^+^CD38^-^) in common with normal stem cells [11]. Subsequently, analogous models have been developed for a number of solid tumors, primarily through the identification of a small population (typically <5%) of tumor cells that were unique both in their expression of a set of specific surface markers as well as their ability to induce phenocopies of their original tumors in xenograft and transplant models [12-19].

Although the cancer stem cell model and the experimental approach to identifying cancer stem cell populations have been replicated across a variety of tissues, the molecular signatures derived from the proliferative cells have varied widely. As yet, the extent to which there exist any molecular fingerprints commonly attributable to multiple types of cancer stem cells remains unclear. While some have been observed to express a subset of the embryonic stem cell-associated genes (*POU5F1*, *NANOG*), the degree to which these trends may be broadly apparent is unknown [20].

The increasing volume of evidence supporting a pervasive connection between cancer and stem cells suggests significant therapeutic implications. As opposed to current therapies that are evaluated based on their ability to reduce the overall size of a tumor, regimens that target cancer stem cells may have more success in preventing long-term recurrence [8]. Molecular signatures that are capable of grading pluripotentiality and proliferative potential represent an important step in designing such regimens and guiding therapeutic procedures.

Indeed, gene expression signatures derived from breast cancer stem cells have been shown to separate patients with early-stage breast cancer into high-risk and low-risk groups [21]. Similarly, gene expression signatures have been used to identify cell-sorted acute myeloid leukemia (AML) samples enriched for leukemic stem cells, and leukemic stem cell expression signatures have been shown to correlate with patient survival [22,23]. Diverse malignant tissue samples have been shown to exhibit a broadly similar trend within a large gene expression database, but no specific connection has been made in this context to stem cell-like activity [24]. However, identifying an unbiased transcriptional measure of 'stemness' conserved across embryonic and adult stem cells, and relating that signature to malignancy, has remained a challenge [6,25,26]. Understanding the mechanisms of tumor proliferation and the relationship of those mechanisms to stem cell pluripotentcy may yield especially important insights into the origins and treatment of germ cell tumors, and embryonal carcinomas in particular, which have been previously demonstrated to express the hallmark ES regulators [27].

Here, we present a comprehensive analysis of a diverse compilation of gene expression samples that reveals a robust multidimensional continuum from ES/induced pluripotent stem cells to fully differentiated tissues. Our results indicate that, within this functional genomic landscape, cancers display a combination of stem cell-like programming and tissue-specific signatures. We derive a shared molecular measure of pluripotentiality that may help bridge the gap between disparate tissue-specific cancer stem cell populations, reflecting their shared proliferative potential. In addition, we demonstrate that our differentiation and pluripotentiality-centric view of gene expression correlates with classical grading systems for a variety of solid tumors, suggesting that our results may form a quantitative axis with practical relevance to personalized medicine.

## Results

### Identifying a stem cell gene set

Our first goal was to identify a set of genes whose expression profiles represent a tightly conserved core of transcriptional programming among stem cells. We call this set of genes the stem cell gene set (SCGS). We derived the SCGS from a high-quality database called Concordia, representing a significant subset of the NCBI's Gene Expression Omnibus (GEO) [28]. Concordia was constructed using a combination of automated textual parsing, human curation and normalization methods (see Materials and methods).

In order to identify a set of genes with highly specific stem cell expression intensities, we used this curated database to identify all of the stem cell samples in our dataset. We then applied a standard signal processing tool, a finite impulse response filter (FIR) [29], to identify those genes with the most highly conserved expression intensities among the stem cell samples. That is, those genes with a range of expression intensities among the stem cell samples that was most distinct from the non-stem cell samples scored the highest (see Materials and methods).

**Figure 1:**
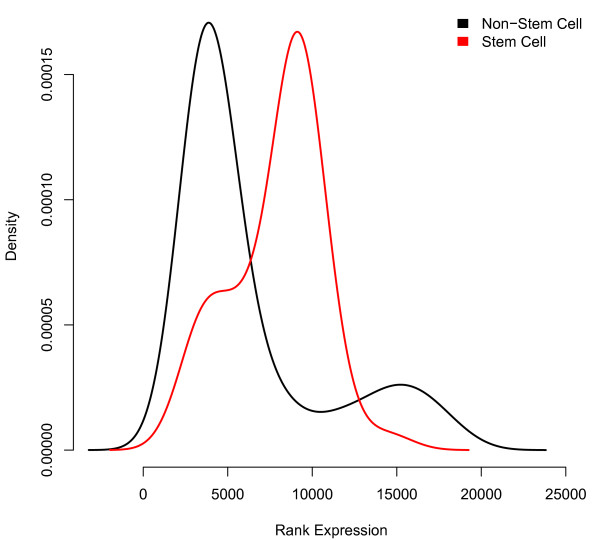
**Distribution of *DBC1 *expression intensities across the entire database**. The distributions of rank-normalized gene expression intensities for gene *DBC1 *are shown for the stem cell samples as well as the non-stem cell samples. The non-stem cell samples clearly exhibit expression both higher and lower than the stem cell samples, while the stem cell samples are relatively specific in their range of expression.

In contrast to a standard *t*-test, this approach does not require us to define a specific 'control' phenotype against which we test for separation, a poorly defined task when comparing against such a diverse database. Moreover, this method identifies genes with expression levels that are highly specific in the stem cell samples, allowing for the diverse population of non-stem cell samples to express these genes at simultaneously higher and lower levels (something for which a *t*-test cannot directly account). For example, the gene *DBC1 *exhibits a highly specific range of expression across the stem cell samples, and ranked highly (among the top 0.5% of all genes) in its ability to localize the stem cell samples by the described method. However, the non-stem cell samples demonstrate both higher and lower expression levels of this gene (Figure 1), causing a standard Student's *t*-test (treating all non-stem cell samples as the control group) to rank this gene at only the 24.6% strongest among all genes.

We verified the ability of the SCGS to capture a nuanced measure of stem cell-like gene expression activity by demonstrating the accurate clustering of a series of developing ES cell populations in mouse (see below). This analysis also shows the concordance between the SCGS transcriptional profile and cellular state of differentiation.

Previous studies have examined the expression patterns of literature-curated gene sets relating to ES-like activity among a variety of malignancies [5]. In contrast, we have constructed a gene set *in silico *that reflects only those transcriptional signals with the greatest ability to localize the stem cell samples within the spectrum of human tissues and diseases.

The 189 genes comprising the SCGS can be found in Tables s1 to s4 in Additional file 1. A variety of FIR thresholds were evaluated according to the ability of the implied gene sets to differentiate between stem cell samples and the other phenotypes in the dataset via an analysis of variance (ANOVA). The genes presented here represent a set capable of simultaneously separating the pluripotent, multipotent, progenitor, malignant and normal samples, while also retaining tissue-specific features (for example, clearly separating normal blood, neural and epithelial tissues). An animation demonstrating the effect of varying the number of top-ranking stem genes included in the SCGS is included in Additional file 2.

### Comparison to previously published stem gene sets

**Figure 2:**
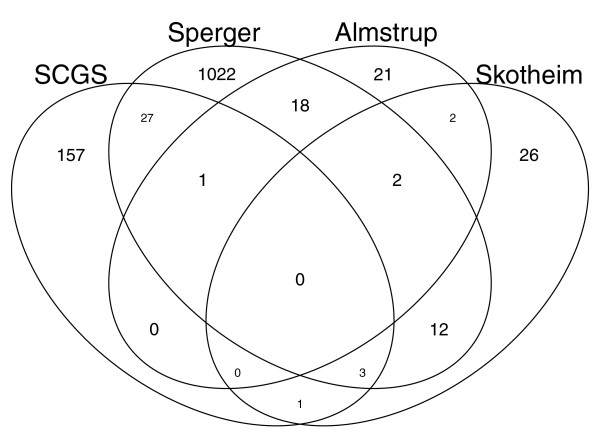
**SCGS overlap with previously identified stem cell genes**. The Venn diagram shows the number of genes in common and distinct to each of the gene sets: Sperger *et al. *[30]; Skotheim *et al. *[31]; Almstrup *et al. *[32].

Several previous attempts have been made to identify the genes responsible for maintaining pluripotency by analyzing the expression patterns of germ cell tumors. Sperger *et al. *[30] performed differential expression analyses between control differentiated cells and ES cells and a variety of germ cell tumors to identify genes with higher expression in pluripotent stem cells. Our approach differs in that we analyzed the expression of only stem cells rather than cultured tumor cell lines. Further, we place no stipulation on differential expression with respect to a fixed control group, but rather focus in on the genes with the greatest ability to characterize the stem cells within a broad spectrum of the human transcriptional landscape. Skotheim *et al. *[31] and Almstrup *et al. *[32] take similar approaches, identifying the genes that characterize an assortment of germ cell tumors. Figure 2 shows the overlap of the SCGS with these previously identified stem gene sets.

### Stem-like signature stratifies a diverse expression database by pluripotentiality and malignancy

Via principal component analysis (PCA), we examined the transcriptional profile of the SCGS across the entire collection of normal tissues, cancers and stem cells assembled from GEO. Performing PCA across only the SCGS genes (including all samples in the data set) allowed us to measure the extent to which the specific transcriptional activity observed in the stem cell population was apparent in each of the other phenotypes.

**Figure 3:**
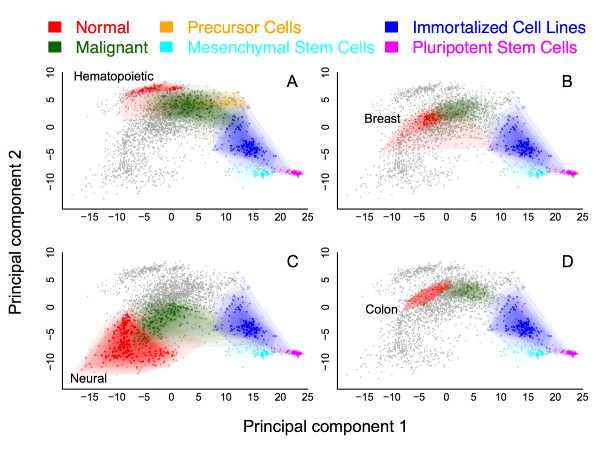
**The spectrum of stem cell-like transcriptional state**. The stem cell signature genes stratify a phenotypically diverse database according to pluripotentiality. Each panel shows the entire expression database plotted on the principal coordinates defined by the stem cell signature genes. PC1 is represented on the x-axis of each plot, while PC2 is on the y-axis. In each plot, the pluripotent stem cells (induced pluripotent stem and ES cells) are clustered on the extreme right-hand side (magenta), followed by mesenchymal stem cells (cyan) and immortalized cell lines (blue). Taken together, the panels demonstrate that, across tissue types, this stem cell signature draws a coherent picture of pluripotentiality and differentiation. While the distinction between the pluripotent stem cells and normal tissues represents the predominant signal (PC1) in the data, the contrast in the expression profiles of hematopoietic and neural tissues apparently defines the second strongest signal (PC2). Even so, both tissues' respective malignancies show a common tendency to exhibit greater stem-like activity, as demonstrated by their closer proximity to the pluripotent stem cell cluster. **(a-d) **Blood (a), breast (b), neural (c) and colon (d) all demonstrate the same enhanced stem-like expression activity among their respective malignancies.

This analysis revealed a striking trend apparent in the first two principal components (PCs) of the gene set; most importantly, PC1 captured a measure of cellular pluripotency, while PC2 reflected the broad transcriptional differences between hematopoietic, neural and epithelial tissues. These trends are demonstrated in Figure 3. Each panel highlights in color the PCA region occupied by a particular normal tissue population (red) and its associated malignancies (green), as well as any related precursor cells (orange), immortalized cell line samples (cyan), multipotent (blue) and pluripotent stem cells (magenta) (PCA was computed jointly across all samples; each cancer is highlighted individually for clarity). The pluripotent stem cells included in this analysis were a combination of both ES cells and induced pluripotent stem cells. The locations of all other samples in the data set are shaded gray to provide context.

The dominant characteristic of PC1 is its ability to separate the pluripotent stem cells from the normal tissue samples (for example, the normal tissues shown in Figure 3 - blood, breast, brain, colon, shaded red, consistently lie on the extreme left side of the plots, whereas the pluripotent stem cells, shaded magenta, lie on the extreme right). Moreover, PC1 apparently reflects a finer-grained continuum of cellular potency: the multipotent stem cells are clustered near the pluripotent stem cells, with the hematopoietic progenitors (the only progenitors in our dataset) slightly farther away (Figure 3a).

Further, the hematopoietic, neural and epithelial cancers (shaded green in Figure 3a-d) contained in our data all clustered directly between the stem cell populations and their associated normal non-malignant samples. This suggests that the SCGS captures a kernel of stem cell-like transcriptional activity that is concurrently apparent in a variety of malignancies. These findings build on previous observations that genes associated with stem cell-like activity demonstrate differential expression in a variety of epithelial cancers with respect to their normal tissue counterparts [6]. Our analysis reveals that stem-like expression profiles are observable not only in epithelial cancers, but also in neural and hematopoietic malignancy as well.

We will use the coordinates of an expression profile's projection into the first principal component of the gene space defined by the SCGS as a relative measure of 'stemness', our stemness index.

The overall landscape of the human transcriptome appears to be organized by a combination of tissue, cell-type and disease-specific features [24]. Previous studies have suggested that the primary factors driving the organization of this landscape are largely attributable to hematopoietic and malignant programming [24]. Our results indicate that while there exists a strong tissue-specific signal, the 'malignancy' signature is more specifically a reflection of the self-renewal and pluripotentiality common to both stem cell populations and heterogeneous tumors.

### Human-derived ES-like transcriptional profile correlates to mouse stem cell differentiation

To verify that our SCGS-derived stemness index captures a quantitative transcriptional measure of differentiation, we used it to examine the expression dynamics of a set of developing mouse ES cells over time [GEO: GSE12550]. This data set consisted of a time-course of differentiating mouse ES cells, with gene expression measured at four time points (ES cells, 4 days of differentiation, 8 days of differentiation and 14 days of differentiation).

Human SCGS gene IDs were mapped to mouse via NCBI's HomoloGene [33]. Human genes that lacked a unique match in mouse were ignored. Expression intensities were processed in an identical manner to the human data (see Materials and methods) and summarized by gene. Again, we computed the dominant variance among the differentiating mouse cells via PCA over the SCGS. We likewise used each mouse ES sample's stemness index (that is, coordinates in the first principal basis) as a summary value of SCGS gene expression activity.

**Figure 4:**
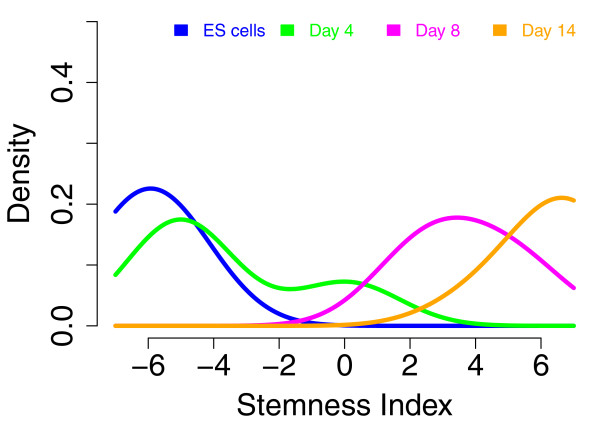
**Distribution of differentiating mouse ES cells over stemness index**. Each curve represents the distribution of stemness index values for a particular time point. This signature collocates the four time points' samples and clearly separates the early and late stages of differentiation.

The dominant expression signal reflected in these genes accurately sorts the samples according to their time point, as shown in Figure 4. This supports the hypothesis that our SCGS-derived stemness index reflects measurable changes in state of differentiation and pluripotentiality, and reflects the fact that the functional genomic mechanisms associated with stem cell activity are at least partially conserved across species [34].

### Stratifying tumor grade

We used the stemness index that we derived from the SCGS to evaluate the transcriptional profiles of several graded tumor data sets. Our goal was to evaluate whether our molecular marker for tissue-agnostic stem cell-like transcriptional activity was representative of poor clinical prognosis. We included four publicly available data sets in this analysis (see Materials and methods). For each data set, we computed the samples' stemness index (via PCA over the SCGS) to identify the dominant differences between the samples within the context of the stem cell genes (Materials and methods).

**Figure 5:**
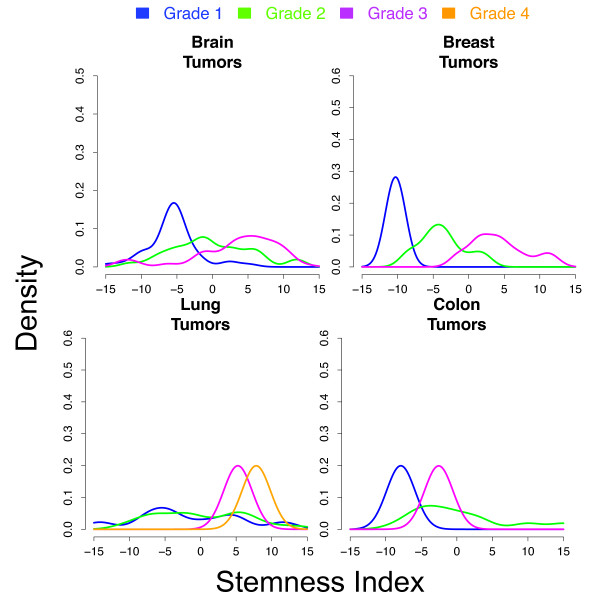
**Stem cell-like activity correlates with tumor grade in various solid malignancies**. Each panel displays the distribution, within the space of the stem cell genes, of graded tumor samples for one particular tissue type. Our stemness index consistently separates high-grade tumors from low grade ones. Based on this transcriptional index, the mid-grade tumors are less well defined.

This analysis revealed that our stemeness index correlates with tumor grade for a variety of primary tissues. Figure 5 shows the distribution of stemness index values for the four tissue types' graded tumor samples. In each case, the transcriptional activity of the SCGS defines a clear separation between the high- and low-graded tumors, while also providing a molecular foundation based on stem-like expression for the clinical difficulty in classifying mid-grade tumors [35,36]. Importantly, such measures should not be considered in isolation, but in concert with standard histopathology, since an aggressive tumor containing a relatively large proportion of normal cells would likely have a low stemness score. As such, these methods may well serve as a 'warning sign' when traditional pathology assigns a low grade, but RNA analysis suggests the tumor is about to turn aggressive.

Recent trends in chemotherapy design have focused not only on regulating cytotoxicity, but also on affecting the differentiation pathways that are apparently impaired in malignant cells. For example, Stegmaier *et al. *[37] have demonstrated the ability of gefitinib to induce myeloid differentiation in both acute myeloid leukemia cell lines as well as patient-derived acute myeloid leukemia blast cells. Indeed, the phenotypic transformation induced by gefitinib was shown to be observable in both cellular morphology and gene expression. The ubiquitous stem cell-like expression patterns described in this paper, as well as those specifically tuned to individual tumor subclasses, may prove useful in screening compounds through the early stages of drug discovery. Understanding the transcriptional changes wrought by these compounds within the context of pluripotentiality and differentiation may be of fundamental value in personalized oncology and therapy selection.

### Functional diversity of the stem cell gene set

Our final goal was to characterize the functional diversity of the genes comprising the SCGS. Hierarchical clustering of these genes' transcriptional activity in a population of pluripotent stem cells revealed four distinct coexpression modules. For each module, we then identified a set of over-enriched Gene Ontology (GO) biological processes [38].

**Figure 6:**
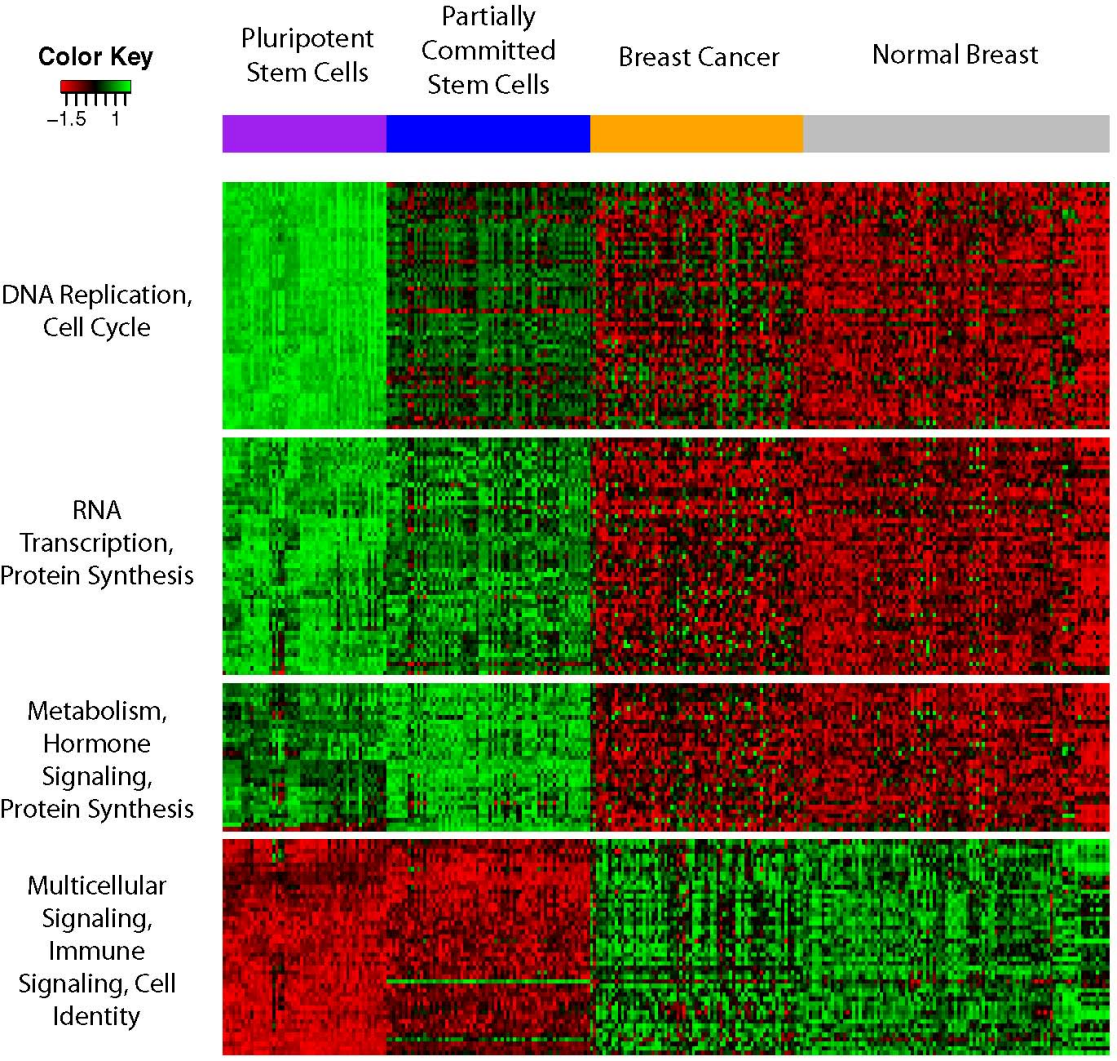
**Expression modules in the SCGS**. Four distinct expression modules (row clusters) are apparent within the stem cell genes. To demonstrate the transcriptome-wide implications of these profiles, this figure displays a series of cell types, ranging from fully differentiated (normal breast), through the associated malignancy, partially committed stem cells, and pluripotent stem cells. Each gene (row) has been independently z-score normalized to improve readability and highlight cluster-specific trends. Biological significance of each cluster was determined by GO analysis (Tables s5 to s8 in Additional file 1). The individual genes represented in each cluster can be found in Tables s1 to s4 in Additional file 1.

To illustrate the gene expression trends apparent within each gene cluster, Figure 6 shows a heatmap of their profiles across pluripotent and partially committed stem cells, as well as malignant and normal breast samples. Genes active in DNA replication, cell cycle regulation and RNA transcription (see Tables s5 and s6 in Additional file 1 for detailed annotations) are most highly expressed in the pluripotent stem cells, and less so, respectively, through increasing levels of cellular differentiation/decreasing pluripotentiality, consistent with prior studies of the dynamics of stem cell cycling and regeneration [25,39]. Genes related to metabolism and hormone signaling (Table s7 in Additional file 1) show peak expression intensity among the partially committed stem cells, while exhibiting low intensity among the fully differentiated tissue and tumor samples. Correspondingly, genes responsible for multicellular signaling and cellular identity (Table s8 in Additional file 1) are most highly expressed in the fully differentiated tissue and malignant samples. Within each functional module, the tumor samples trend away from the respective normal tissue, echoing stem cell-like transcriptional activity.

## Conclusions

We have demonstrated conserved stem cell-like transcriptional activity across a wide variety of hematopoietic and solid cancers through a comprehensive molecular survey of malignancy, pluripotent stem cells and normal tissues. Our findings echo several recent developments in the cancer stem cell debate. In particular, our results highlight transcriptional evidence that, despite individual tissue-specific characteristics, a wide range of cancers share a common set of transcriptional mechanisms with each other, as well as pluripotent and multipotent stem cells.

While a large volume of evidence indicates that only a small number of tumor cells are capable of self-renewal, controversy remains as to the exact origin of these cells. The hierarchical cancer stem cell hypothesis suggests that these cells arise from normal pluripotent or multipotent stem cells that have lost the ability to regulate their proliferative activity. Under this model, the phenotypic diversity observed in many tumors is viewed as the result of this defective stem cell population mismanaging the process of normal organogenesis. Alternatively, the stochastic model of tumorigenesis suggests that proliferative tumor cells arise from normal fully differentiated or committed progenitor cells that acquire the ability to self renew, and that tumor cell phenotype variation is the result of these mutated cells differentiating in a random fashion [40].

Regardless of the origin of proliferative tumor cells, our results indicate that there is a high degree of stem cell-specific gene expression programming observable in heterogeneous tumor samples. Our data indicate the need for more detailed transcriptional assays comparing proliferative tumor cells to both ES/induced pluripotent stem cells and bulk heterogeneous tumor cells, as well as normal tissue cells. Our data suggest the hypothesis that the gene expression patterns observed in heterogeneous tumor samples may be due to the effect of a small population of cancer stem cells in combination with a large number of partially differentiated cells. It is plausible that, while the partially differentiated mass of the tumor behaves transcriptionally similarly to healthy tissue, the small population of proliferative tumor cells pushes the observation of the aggregate mRNA back along the spectrum of stem cell-like activity identified in this paper.

We have demonstrated a specific transcriptional signal that is shared among a wide variety of solid and hematopoietic cancers. Moreover, when considered from a transcriptome-wide perspective, this signal is indicative of stem cell-like activity. We have shown how these gene expression patterns are most strongly associated with embryonic and induced pluripotent stem cells, and are successively less apparent in multipotent stem cells, malignancies, and fully differentiated tissues, respectively. In addition, the genes that comprise this signal also reveal a stratification of solid tumors that correlates strongly with classical grading systems.

## Materials and methods

### Concordia, a large phenotypically diverse gene expression database

The Concordia database contains 3,209 Affymetrix HGU133+ 2.0 gene expression array samples (all from human tissue or cultured human cell lines) extracted from NCBI's GEO. A full description of the techniques used to assemble this database have been previously described [41], and the curated phenotype data are available for public download at the Concordia database web site [42], including all of the non-malignant, malignant and stem cell samples, less the external graded tumor sets that were used to verify the SCGS signal's relationship to solid tumor histology. The following two sections briefly describe the Concordia database.

### Using UMLS annotation to associate each sample with its relevant phenotypes

We constructed a database representing a subset (3,209 samples) of NCBI's GEO [28,33] that contained a combination of samples derived from normal tissues, immortalized cell lines, a variety of cancers, and an assortment of pluripotent and partially committed stem cells. In order to generate high-quality, systematic phenotype annotations for this dataset, the GEO text descriptions relating to each sample (including title, description, and source fields) were mapped into the Unified Medical Language System's (UMLS) [43] ontology of biological and medical concepts. This was done using a combination of natural language processing (NLP) software and hand validation to remove spurious associations.

NLP was performed by the Java implementation of the National Library of Medicine's (NLM) MetaMap program, MMTx [44]. A custom UMLS thesaurus was generated using NLM's MetaMorphosys program that contained the concepts and relationships from the UMLS, MeSH, and SNOMED ontologies.

These automated annotations were then verified by hand so as to remove false positives. Using custom-built software, these associations were propagated through the ontology's hierarchy, allowing us to identify all samples related to phenotypes of arbitrary specificity.

### Normalizing the gene expression samples

The expression data for the samples in our dataset were obtained from their respective GEO CEL files, which were MAS 5.0 [45] normalized via R's BioConductor package [46,47]. The resulting probe set intensities were averaged into 20,252 unique gene-centric values, and then rank normalized to improve cross-data series comparability. All calculations were performed in the R statistical environment, employing the BioConductors suite.

### Additional expression data

In addition to the Concordia gene expression data, several additional GEO data sets were used to analyze the SCGS signal's relationship to histological tumor grade. These are: a series of graded glioma tumor samples [GEO: GSE4290]; a series of graded tumor samples from core needle biopsies of breast cancer patients, including a variety of estrogen receptor-positive and -negative and progesterone receptor-positive and -negative phenotypes [GEO: GSE23593]; a set of graded lung tumors, including a variety of squamous and adenocarcinoma samples [GEO: GSE18842]; and a set of graded colon tumors [GEO: GSE17537].

### Using FIR to identify genes that characterize pluripotent stem cells

We sought to associate with each gene a measure of how well conserved its expression intensity was over the stem cell samples. Rather than seeking a strict measure of constitutive over- or under-expression of the gene among the stem cell population, our goal was instead to identify individual genes that tightly cluster the stem cell population anywhere along the spectrum of expression intensities.

Here, we employ a signal-processing tool, the FIR [29]. The input to this procedure is a list of all of the expression samples, sorted according to their intensity for a particular gene. The filter then applies a 'sliding window' to the list and outputs, at each window position, the proportion of stem cell samples within the frame. The maximal value of this sliding window at any position in the list is then taken as that gene's score. We use a window equal in size to the total number of stem cell samples in the database, so the interpretation of the filter's maximal output is intuitive: if we are looking to find all of the stem cell samples within one window frame, what is the greatest fraction that we can localize, given the expression values for this gene across the entire database? Genes with the highest scores are those with most specific stem cell expression intensities.

Binomial *P*-values (*k *= number of stem cell samples in a given window frame; *n *= window frame size; *p *= proportion of stem cell samples in the entire database) are reported along with these scores.

**Figure 7:**
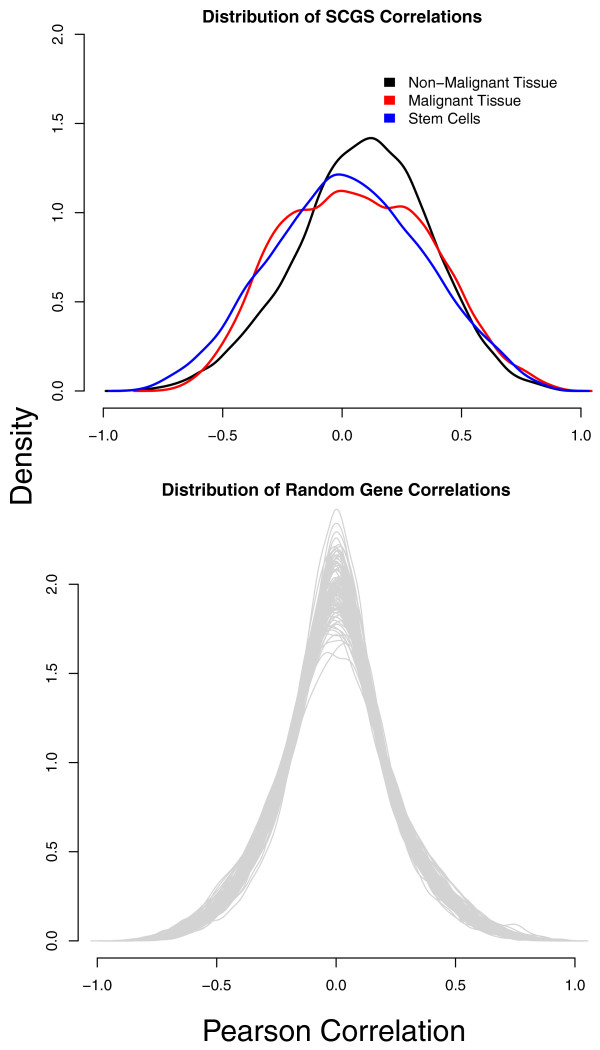
**Inter-gene SCGS correlation across various sample types**. The distribution of SCGS gene-gene correlations are shown in the top panel independently for the non-malignant, malignant and stem cell samples contained in the database. The distribution of gene-gene correlations for 1,000 random sets of genes equal in size to the SCGS is shown in the bottom panel.

To ensure that the method was not simply selecting genes that are all highly correlated with each other across the entire database, we computed the distribution of SCGS Pearson correlation coefficients over the stem cell samples, malignant tissue samples and non-malignant tissue samples independently, then compared those distributions to 1,000 random sets of genes equal in size to the SCGS. Only the non-malignant tissue samples show a positive location shift (Figure 7).

### Summarizing expression signals across a group of genes via PCA

In order to capture a continuous measure of SCGS activity, we applied PCA [48]. The basis vector associated with the largest eigenvalue of the gene-gene covariance matrix captures the dominant coordinated signal present within the gene set. By projecting each sample's observed expression intensity onto this basis, we compute a summary value describing the sample's affinity for a stem cell-like gene expression profile.

### Measuring tumor grade along the continuum of stem-like expression

We identified four independent data series containing expression profiles for graded tumors of various tissue types in GEO ([GEO: GSE4290], [GEO: GSE23593], [GEO: GSE17537], [GEO: GSE18842]) on Affymetrix HGU 133+ 2.0. Each series was pre-processed (MAS5.0 normalized, summarized) as previously described. Within each series, the SCGS summary values were computed, again, via PCA over this gene set, allowing us to associate a value with each sample indicating its relative stem-like expression activity.

### SCGS clustering and GO enrichment

The SCGS was clustered using the gplots package for R. Genes were individually quantile normalized to improve readability of the resulting figures. GO biological process enrichment calculations were performed on the individual clusters using the GOstats BioConductor library [38,49].

### Data access

All microarray samples included in these analyses are publicly available via the GEO. Accession IDs for each sample are included in Additional file 1, and curated, machine-readable phenotype information for those samples is available at the Concordia database web site [42].

## Abbreviations

ANOVA: Analysis of Variance; ES, embryonic stem; FIR: finite impulse response filter; GEO: Gene Expression Omnibus; GO: Gene Ontology; NLM: National Library of Medicine; NLP: natural language processing; PC: principal component; PCA: principal component analysis; SCGS: stem cell gene set; UMLS: Unified Medical Language System.

## Competing interests

The authors declare that they have no competing interests.

## Authors' contributions

NP contributed to the study design, contributed to data set curation, performed statistical analyses, and wrote the manuscript. PS assisted in data curation, study design and manuscript preparation. IK and BB lead the study design, advised on statistical analyses, and assisted in manuscript preparation. All authors have read and approved the manuscript for publication.

## Supplementary Material

Additional file 1 **Supplementary tables**. Tables s1 to s4: genes in the SCGS, organized by the functional module to which they belong. Tables s5 to s8: GO enrichment statistics for each functional module in the SCGS. The file also includes a complete listing of all of the GEO sample identifiers for the microarray data comprising our database. Click here for file

Additional file 2 **This file contains an animation demonstrating the effect of varying the FIR score threshold for including genes in the SCGS**. For each possible number of top-scoring stem genes from 3-502 (displayed at the top of the animation frame), we project all of the samples in the database into the first two PCs of gene space (panel on top right), and highlight in color six relevant phenotypes (as in Figure 3): embryonic/induced pluripotent stem cells in magenta; mesenchymal stem cells in cyan; immortalized cell line samples in blue; blood precursor cells in orange; leukemia samples in green; normal blood in red. The panel below the PCA scatter plot shows the distribution of stemness index values (PC1 projection coordinates) for each highlighted phenotype. The plot on the left of the frame shows the analysis of variance (ANOVA) score (including all highlighted phenotypes) for the clustering defined by the current stemness index highlighted by a magenta dot on the curve showing all ANOVA scores for all of the depicted FIR thresholds. Higher ANOVA scores indicate better multi-way separation of the individual phenotypes along the stemness index. ANOVA was calculated and all plots were generated in the R statistical environment [46,47]. Click here for file

## References

[B1] Rivera MN Haber DA Wilms' tumour: connecting tumorigenesis and organ development in the kidney. Nat Rev Cancer 2005 5 699 712 10.1038/nrc1696 16110318

[B2] Scotting PJ Walker DA Perilongo G Childhood solid tumours: a developmental disorder. Nat Rev Cancer 2005 5 481 488 10.1038/nrc1633 15905853

[B3] Stiewe T The p53 family in differentiation and tumorigenesis. Nat Rev Cancer 2007 7 165 168 10.1038/nrc2072 17332760

[B4] Naxerova K Bult CJ Peaston A Fancher K Knowles BB Kasif S Kohane IS Analysis of gene expression in a developmental context emphasizes distinct biological leitmotifs in human cancers. Genome Biol 2008 9 R108 10.1186/gb-2008-9-7-r108 18611264 PMC2530866

[B5] Ben-Porath I Thomson MW Carey VJ Ge R Bell GW Regev A Weinberg RA An embryonic stem cell-like gene expression signature in poorly differentiated aggressive human tumors. Nat Genet 2008 40 499 507 10.1038/ng.127 18443585 PMC2912221

[B6] Wong DJ Liu H Ridky TW Cassarino D Segal E Chang HY Module map of stem cell genes guides creation of epithelial cancer stem cells. Cell Stem Cell 2008 2 333 344 10.1016/j.stem.2008.02.009 18397753 PMC2628721

[B7] Li P Zon LI Resolving the controversy about N-cadherin and hematopoietic stem cells. Cell Stem Cell 2010 6 199 202 10.1016/j.stem.2010.02.007 20207222

[B8] Visvader JE Lindeman GJ Cancer stem cells in solid tumours: accumulating evidence and unresolved questions. Nat Rev Cancer 2008 8 755 768 10.1038/nrc2499 18784658

[B9] Heppner GH Miller BE Tumor heterogeneity: biological implications and therapeutic consequences. Cancer Metastasis Rev 1983 2 5 23 10.1007/BF00046903 6616442

[B10] Dontu G Al-Hajj M Abdallah WM Clarke MF Wicha MS Stem cells in normal breast development and breast cancer. Cell Prolif 2003 36 Suppl 1 59 72 14521516 10.1046/j.1365-2184.36.s.1.6.x PMC6495427

[B11] Fialkow PJ Stem cell origin of human myeloid blood cell neoplasms. Verhandlungen der Deutschen Gesellschaft für Pathologie 1990 74 43 47 1708632

[B12] Singh SK Clarke ID Terasaki M Bonn VE Hawkins C Squire J Dirks PB Identification of a cancer stem cell in human brain tumors. Cancer Res 2003 63 5821 5828 14522905

[B13] Al-Hajj M Wicha MS Benito-Hernandez A Morrison SJ Clarke MF Prospective identification of tumorigenic breast cancer cells. Proc Natl Acad Sci USA 2003 100 3983 3988 10.1073/pnas.0530291100 12629218 PMC153034

[B14] Fang D Nguyen TK Leishear K Finko R Kulp AN Hotz S Van Belle PA Xu X Elder DE Herlyn M A tumorigenic subpopulation with stem cell properties in melanomas. Cancer Res 2005 65 9328 9337 10.1158/0008-5472.CAN-05-1343 16230395

[B15] Bapat SA Mali AM Koppikar CB Kurrey NK Stem and progenitor-like cells contribute to the aggressive behavior of human epithelial ovarian cancer. Cancer Res 2005 65 3025 3029 15833827 10.1158/0008-5472.CAN-04-3931

[B16] Collins AT Berry PA Hyde C Stower MJ Maitland NJ Prospective identification of tumorigenic prostate cancer stem cells. Cancer Res 2005 65 10946 10951 10.1158/0008-5472.CAN-05-2018 16322242

[B17] Gibbs CP Kukekov VG Reith JD Tchigrinova O Suslov ON Scott EW Ghivizzani SC Ignatova TN Steindler DA Stem-like cells in bone sarcomas: implications for tumorigenesis. Neoplasia 2005 7 967 976 10.1593/neo.05394 16331882 PMC1502023

[B18] Ricci-Vitiani L Lombardi DG Pilozzi E Biffoni M Todaro M Peschle C De Maria R Identification and expansion of human colon-cancer-initiating cells. Nature 2007 445 111 115 10.1038/nature05384 17122771

[B19] Lobo NA Shimono Y Qian D Clarke MF The biology of cancer stem cells. Annu Rev Cell Dev Biol 2007 23 675 699 10.1146/annurev.cellbio.22.010305.104154 17645413

[B20] Yu J Vodyanik MA Smuga-Otto K Antosiewicz-Bourget J Frane JL Tian S Nie J Jonsdottir GA Ruotti V Stewart R Slukvin II Thomson JA Induced pluripotent stem cell lines derived from human somatic cells. Science 2007 318 1917 1920 10.1126/science.1151526 18029452

[B21] Liu R Wang X Chen GY Dalerba P Gurney A Hoey T Sherlock G Lewicki J Shedden K Clarke MF The prognostic role of a gene signature from tumorigenic breast-cancer cells. N Engl J Med 2007 356 217 226 10.1056/NEJMoa063994 17229949

[B22] Gentles AJ Plevritis SK Majeti R Alizadeh AA Association of a leukemic stem cell gene expression signature with clinical outcomes in acute myeloid leukemia. JAMA 2010 304 2706 2715 10.1001/jama.2010.1862 21177505 PMC4089862

[B23] Eppert K Takenaka K Lechman ER Waldron L Nilsson B van Galen P Metzeler KH Poeppl A Ling V Beyene J Canty AJ Danska JS Bohlander SK Buske C Minden MD Golub TR Jurisica I Ebert BL Dick JE Stem cell gene expression programs influence clinical outcome in human leukemia. Nat Med 2011 17 1086 1093 10.1038/nm.2415 21873988

[B24] Lukk M Kapushesky M Nikkilä J Parkinson H Goncalves A Huber W Ukkonen E Brazma A A global map of human gene expression. Nat Biotechnol 2010 28 322 324 10.1038/nbt0410-322 20379172 PMC2974261

[B25] Ramalho-Santos M Yoon S Matsuzaki Y Mulligan RC Melton DA "Stemness": transcriptional profiling of embryonic and adult stem cells. Science 2002 298 597 600 10.1126/science.1072530 12228720

[B26] Fortunel NO Otu HH Ng H-H Chen J Mu X Chevassut T Li X Joseph M Bailey C Hatzfeld JA Hatzfeld A Usta F Vega VB Long PM Libermann TA Lim B Comment on " 'Stemness': transcriptional profiling of embryonic and adult stem cells" and "a stem cell molecular signature". Science 2003 302 393 author reply 393 14563990 10.1126/science.1086384

[B27] Gillis AJM Stoop H Biermann K van Gurp RJHLM Swartzman E Cribbes S Ferlinz A Shannon M Oosterhuis JW Looijenga LHJ Expression and interdependencies of pluripotency factors LIN28, OCT3/4, NANOG and SOX2 in human testicular germ cells and tumours of the testis. Int J Androl 2011 34 e160 74 10.1111/j.1365-2605.2011.01148.x 21631526

[B28] Barrett T Troup DB Wilhite SE Ledoux P Evangelista C Kim IF Tomashevsky M Marshall KA Phillippy KH Sherman PM Muertter RN Holko M Ayanbule O Yefanov A Soboleva A NCBI GEO: archive for functional genomics data sets--10 years on. Nucleic Acids Res 2011 39 D1005 1010 10.1093/nar/gkq1184 21097893 PMC3013736

[B29] McClellan JH Schafer RW Yoder MA DSP First: a Multimedia Approach 1998 Prentice Hall

[B30] Sperger JM Chen X Draper JS Antosiewicz JE Chon CH Jones SB Brooks JD Andrews PW Brown PO Thomson JA Gene expression patterns in human embryonic stem cells and human pluripotent germ cell tumors. Proc Natl Acad Sci USA 2003 100 13350 13355 10.1073/pnas.2235735100 14595015 PMC263817

[B31] Skotheim RI Lind GE Monni O Nesland JM Abeler VM Fosså SD Duale N Brunborg G Kallioniemi O Andrews PW Lothe RA Differentiation of human embryonal carcinomas in vitro and in vivo reveals expression profiles relevant to normal development. Cancer Res 2005 65 5588 5598 10.1158/0008-5472.CAN-05-0153 15994931

[B32] Almstrup K Hoei-Hansen CE Wirkner U Blake J Schwager C Ansorge W Nielsen JE Skakkebaek NE Rajpert-De Meyts E Leffers H Embryonic stem cell-like features of testicular carcinoma in situ revealed by genome-wide gene expression profiling. Cancer Res 2004 64 4736 4743 10.1158/0008-5472.CAN-04-0679 15256440

[B33] Sayers EW Barrett T Benson DA Bolton E Bryant SH Canese K Chetvernin V Church DM DiCuccio M Federhen S Feolo M Fingerman IM Geer LY Helmberg W Kapustin Y Landsman D Lipman DJ Lu Z Madden TL Madej T Maglott DR Marchler-Bauer A Miller V Mizrachi I Ostell J Panchenko A Phan L Pruitt KD Schuler GD Sequeira E Database resources of the National Center for Biotechnology Information. Nucleic Acids Res 2011 39 D38 51 10.1093/nar/gkq1172 21097890 PMC3013733

[B34] Cai J Xie D Fan Z Chipperfield H Marden J Wong WH Zhong S Modeling co-expression across species for complex traits: insights to the difference of human and mouse embryonic stem cells. PLoS Comp Biol 2010 6 e1000707 10.1371/journal.pcbi.1000707 PMC2837392 20300647

[B35] Tonn JC Westphal M Neuro-oncology of CNS tumors 2006 Springer Verlag

[B36] Fuller GN Mircean C Tabus I Taylor E Sawaya R Bruner JM Shmulevich I Zhang W Molecular voting for glioma classification reflecting heterogeneity in the continuum of cancer progression. Oncol Rep 2005 14 651 656 16077969

[B37] Stegmaier K Corsello SM Ross KN Wong JS Deangelo DJ Golub TR Gefitinib induces myeloid differentiation of acute myeloid leukemia. Blood 2005 106 2841 2848 10.1182/blood-2005-02-0488 15998836 PMC1895296

[B38] Ashburner M Ball CA Blake JA Botstein D Butler H Cherry JM Davis AP Dolinski K Dwight SS Eppig JT Harris MA Hill DP Issel-Tarver L Kasarskis A Lewis S Matese JC Richardson JE Ringwald M Rubin GM Sherlock G Gene ontology: tool for the unification of biology. The Gene Ontology Consortium. Nat Genet 2000 25 25 29 10.1038/75556 10802651 PMC3037419

[B39] Takizawa H Regoes RR Boddupalli CS Bonhoeffer S Manz MG Dynamic variation in cycling of hematopoietic stem cells in steady state and inflammation. J Exp Med 2011 208 273 284 10.1084/jem.20101643 21300914 PMC3039863

[B40] Gupta PB Fillmore CM Jiang G Shapira SD Tao K Kuperwasser C Lander ES Stochastic state transitions give rise to phenotypic equilibrium in populations of cancer cells. Cell 2011 146 633 644 10.1016/j.cell.2011.07.026 21854987

[B41] Schmid PR Palmer NP Kohane IS Berger B Making sense out of massive data by going beyond differential expression. Proc Natl Acad Sci USA 2012 109 5594 5599 10.1073/pnas.1118792109 22447773 PMC3326474

[B42] Concordia. http://concordia.csail.mit.edu

[B43] Bodenreider O The Unified Medical Language System (UMLS): integrating biomedical terminology. Nucleic Acids Res 2004 32 D267 270 10.1093/nar/gkh061 14681409 PMC308795

[B44] Osborne JD Lin S Zhu L Kibbe WA Mining biomedical data using MetaMap Transfer (MMtx) and the Unified Medical Language System (UMLS). Methods Mol Biol 2007 408 153 169 10.1007/978-1-59745-547-3_9 18314582

[B45] Affymetrix Affymetrix Microarray Suite User Guide http://www.affymetrix.com/support/technical/manuals.affx

[B46] R Development Core Team R: A Language and Environment for Statistical Computing. Vienna, Austria 2007

[B47] Gentleman RC Carey VJ Bates DM Bolstad B Dettling M Dudoit S Ellis B Gautier L Ge Y Gentry J Hornik K Hothorn T Huber W Iacus S Irizarry R Leisch F Li C Maechler M Rossini AJ Sawitzki G Smith C Smyth G Tierney L Yang JYH Zhang J Bioconductor: open software development for computational biology and bioinformatics. Genome Biol 2004 5 R80 10.1186/gb-2004-5-10-r80 15461798 PMC545600

[B48] Kohane IS Butte AJ Kho A Microarrays for an Integrative Genomics 2002 Cambridge, MA, USA: MIT Press

[B49] Falcon S Gentleman R Using GOstats to test gene lists for GO term association. Bioinformatics 2007 23 257 258 10.1093/bioinformatics/btl567 17098774

