# Supplementary material for: A gene expression profile of stem cell pluripotentiality and differentiation is conserved across diverse solid and hematopoietic cancers
Source: Genome Biol. 2012 Aug 21;13(8):R71. doi: 10.1186/gb-2012-13-8-r71 (PMC3491371; doi:10.1186/gb-2012-13-8-r71)
Supplement: Additional file 1 — Supplementary tables. Tables s1 to s4: genes in the SCGS, organized by the functional module to which they belong. Tables s5 to s8: GO enrichment statistics for each functional module in the SCGS. The file also includes a complete listing of all of the GEO sample identifiers for the microarray data comprising our database. [file gb-2012-13-8-r71-S1.PDF]

Supplemental Material

Table s1: SCGS genes in the DNA replication / cell cycle module. The FIR score, percentile, and Bonferroni-corrected p-value (see Methods) are reported for each gene in the set.

Gene Name	Gene ID	Score	Binomial p-value	Percentile
DNMT3B	1789	0.508379888	2.94E-61	0.00296267
MCM6	4175	0.51396648	1.62E-62	0.002666403
CDC25A	993	0.525139665	4.62E-65	0.002024491
PFAS	5198	0.525139665	4.62E-65	0.002024491
MCM4	4173	0.452513966	3.30E-49	0.008641122
XRCC5	7520	0.480446927	4.11E-55	0.005184673
HAUS6	54801	0.458100559	2.28E-50	0.007406676
TET1	80312	0.458100559	2.28E-50	0.007406676
IGF2BP1	10642	0.541899441	5.95E-69	0.001580091
PLAA	9373	0.469273743	1.01E-52	0.006270986
DEPDC1B	55789	0.458100559	2.28E-50	0.007406676
TEX10	54881	0.458100559	2.28E-50	0.007406676
CCDC99	54908	0.558659218	6.26E-73	0.001234446
MSH2	4436	0.480446927	4.11E-55	0.005184673
BUB1B	701	0.480446927	4.11E-55	0.005184673
MSH6	2956	0.463687151	1.53E-51	0.007011653
DLGAP5	9787	0.491620112	1.53E-57	0.004147738
SKIV2L2	23517	0.469273743	1.01E-52	0.006270986
CENPE	1062	0.474860335	6.52E-54	0.005629074
CHEK2	11200	0.525139665	4.62E-65	0.002024491
SOHLH2	54937	0.603351955	5.68E-84	0.000345645
CCNB1	891	0.458100559	2.28E-50	0.007406676
RRAS2	22800	0.581005587	2.26E-78	0.000641912
PRIM1	5557	0.474860335	6.52E-54	0.005629074
PAICS	10606	0.469273743	1.01E-52	0.006270986
CCNA2	890	0.497206704	9.02E-59	0.003703338
CPSF3	51692	0.474860335	6.52E-54	0.005629074
NUSAP1	51203	0.469273743	1.01E-52	0.006270986
LIN28B	389421	0.502793296	5.21E-60	0.00320956
IPO5	3843	0.525139665	4.62E-65	0.002024491
KIF11	3832	0.48603352	2.54E-56	0.004690895
BMPR1A	657	0.452513966	3.30E-49	0.008641122
NDC80	10403	0.491620112	1.53E-57	0.004147738
BCAT1	586	0.519553073	8.75E-64	0.002419514
CCNG1	900	0.508379888	2.94E-61	0.00296267
ZNF788	388507	0.469273743	1.01E-52	0.006270986

ASCC3	10973	0.452513966	3.30E-49	0.008641122
FANCB	2187	0.458100559	2.28E-50	0.007406676
MCM10	55388	0.525139665	4.62E-65	0.002024491
HMGA2	8091	0.469273743	1.01E-52	0.006270986
SKP2	6502	0.469273743	1.01E-52	0.006270986
TRIM24	8805	0.541899441	5.95E-69	0.001580091
ORC1	4998	0.480446927	4.11E-55	0.005184673
HDAC2	3066	0.458100559	2.28E-50	0.007406676
HESX1	8820	0.480446927	4.11E-55	0.005184673
C1orf135	79000	0.51396648	1.62E-62	0.002666403
INHBE	83729	0.497206704	9.02E-59	0.003703338
MIS18A	54069	0.463687151	1.53E-51	0.007011653
DCUN1D5	84259	0.463687151	1.53E-51	0.007011653
POLE2	5427	0.48603352	2.54E-56	0.004690895
MRPL3	11222	0.469273743	1.01E-52	0.006270986
CENPH	64946	0.463687151	1.53E-51	0.007011653
MYCN	4613	0.458100559	2.28E-50	0.007406676
HAUS1	115106	0.474860335	6.52E-54	0.005629074
GDF3	9573	0.458100559	2.28E-50	0.007406676

Table s2: SCGS genes in the RNA transcription / protein synthesis module. The FIR score, percentile, and Bonferroni-corrected p-value (see Methods) are reported for each gene in the set.

Gene Name	Gene ID	Score	Binomial p-value	Percentile
TBCE	6905	0.491620112	1.53E-57	0.004147738
RIOK2	55781	0.597765363	1.48E-82	0.000395023
BCKDHB	594	0.458100559	2.28E-50	0.007406676
RAD1	5810	0.458100559	2.28E-50	0.007406676
NREP	9315	0.458100559	2.28E-50	0.007406676
ADH5	128	0.648044693	1.16E-95	0.000197511
PLRG1	5356	0.519553073	8.75E-64	0.002419514
ROR1	4919	0.670391061	9.24E-102	4.94E-05
RAB3B	5865	0.553072626	1.36E-71	0.001431957
LOC285431	285431	0.491620112	1.53E-57	0.004147738
DBC1	1620	0.48603352	2.54E-56	0.004690895
KIF23	9493	0.452513966	3.30E-49	0.008641122
DIAPH3	81624	0.502793296	5.21E-60	0.00320956
GNL2	29889	0.491620112	1.53E-57	0.004147738
FGF2	2247	0.681564246	7.10E-105	0
TARDBP	23435	0.458100559	2.28E-50	0.007406676
NMNAT2	23057	0.452513966	3.30E-49	0.008641122
ZNF167	55888	0.491620112	1.53E-57	0.004147738

KIF20A	10112	0.463687151	1.53E-51	0.007011653
CENPI	2491	0.480446927	4.11E-55	0.005184673
DDX1	1653	0.469273743	1.01E-52	0.006270986
XXYLT1	152002	0.525139665	4.62E-65	0.002024491
GPR176	11245	0.664804469	3.21E-100	9.88E-05
FBXO22	26263	0.469273743	1.01E-52	0.006270986
BBS9	27241	0.51396648	1.62E-62	0.002666403
C14orf166	51637	0.541899441	5.95E-69	0.001580091
BOD1	91272	0.519553073	8.75E-64	0.002419514
CDC123	8872	0.469273743	1.01E-52	0.006270986
SNRPD3	6634	0.502793296	5.21E-60	0.00320956
FAM118B	79607	0.56424581	2.82E-74	0.000987557
DPH3	285381	0.474860335	6.52E-54	0.005629074
EIF2B3	8891	0.469273743	1.01E-52	0.006270986
KDELC1	79070	0.586592179	9.33E-80	0.000543156
RPF2	84154	0.458100559	2.28E-50	0.007406676
APLP1	333	0.474860335	6.52E-54	0.005629074
DACT1	51339	0.536312849	1.20E-67	0.001777602
PDHB	5162	0.586592179	9.33E-80	0.000543156
C14orf119	55017	0.575418994	5.37E-77	0.000790045
DTD1	92675	0.469273743	1.01E-52	0.006270986
SAMM50	25813	0.497206704	9.02E-59	0.003703338
CCL26	10344	0.491620112	1.53E-57	0.004147738
C4orf52	389203	0.458100559	2.28E-50	0.007406676
CCDC90B	60492	0.458100559	2.28E-50	0.007406676
MED20	9477	0.56424581	2.82E-74	0.000987557
UTP6	55813	0.469273743	1.01E-52	0.006270986
RARS2	57038	0.458100559	2.28E-50	0.007406676
KIAA0020	9933	0.474860335	6.52E-54	0.005629074
ARMCX2	9823	0.569832402	1.25E-75	0.000839423
RARS	5917	0.491620112	1.53E-57	0.004147738
MTHFD2	10797	0.469273743	1.01E-52	0.006270986
DHX15	1665	0.452513966	3.30E-49	0.008641122
HTR7	3363	0.558659218	6.26E-73	0.001234446
HIST1H4C	8364	0.48603352	2.54E-56	0.004690895

Table s3: SCGS genes in the metabolism / hormone signaling / protein synthesis module. The FIR score, percentile, and Bonferroni-corrected p-value (see Methods) are reported for each gene in the set.

Gene Name	Gene ID	Score	Binomial p-value	Percentile
MTHFD1L	25902	0.541899441	5.95E-69	0.001580091

ARMC9	80210	0.569832402	1.25E-75	0.000839423
XPOT	11260	0.51396648	1.62E-62	0.002666403
IARS	3376	0.497206704	9.02E-59	0.003703338
HDX	139324	0.56424581	2.82E-74	0.000987557
ACTRT3	84517	0.530726257	2.39E-66	0.001925736
ERCC2	2068	0.458100559	2.28E-50	0.007406676
TBC1D16	125058	0.452513966	3.30E-49	0.008641122
GARS	2617	0.497206704	9.02E-59	0.003703338
KIF7	374654	0.61452514	7.83E-87	0.000296267
UBE2K	3093	0.508379888	2.94E-61	0.00296267
SLC25A3	5250	0.48603352	2.54E-56	0.004690895
ICMT	23463	0.530726257	2.39E-66	0.001925736
UGGT2	55757	0.48603352	2.54E-56	0.004690895
ATP11C	286410	0.48603352	2.54E-56	0.004690895
SLC24A1	9187	0.497206704	9.02E-59	0.003703338
EIF2AK4	440275	0.474860335	6.52E-54	0.005629074
GPX8	493869	0.491620112	1.53E-57	0.004147738
ALX1	8092	0.51396648	1.62E-62	0.002666403
OSTC	58505	0.525139665	4.62E-65	0.002024491
TRPC4	7223	0.458100559	2.28E-50	0.007406676
HAS2	3037	0.51396648	1.62E-62	0.002666403
FZD2	2535	0.452513966	3.30E-49	0.008641122
TRNT1	51095	0.519553073	8.75E-64	0.002419514
MMADHC	27249	0.536312849	1.20E-67	0.001777602
SNX8	29886	0.502793296	5.21E-60	0.00320956
CDH6	1004	0.458100559	2.28E-50	0.007406676
HAT1	8520	0.458100559	2.28E-50	0.007406676
SEC11A	23478	0.519553073	8.75E-64	0.002419514
DIMT1	27292	0.452513966	3.30E-49	0.008641122
TM2D2	83877	0.452513966	3.30E-49	0.008641122
FST	10468	0.536312849	1.20E-67	0.001777602
GBE1	2632	0.480446927	4.11E-55	0.005184673

Table s4: SCGS genes in the multicellular signaling / immune signaling / cell identity module. The FIR score, percentile, and Bonferroni-corrected p-value (see Methods) are reported for each gene in the set.

Gene Name	Gene ID	Score	Binomial p-value	Percentile
NA	80047	0.452513966	3.30E-49	0.008641122
MLL3	58508	0.508379888	2.94E-61	0.00296267
MXI1	4601	0.480446927	4.11E-55	0.005184673
FKSG49	400949	0.569832402	1.25E-75	0.000839423

FAM185BP	641808	0.48603352	2.54E-56	0.004690895
ARRB2	409	0.56424581	2.82E-74	0.000987557
SMARCC2	6601	0.497206704	9.02E-59	0.003703338
WASH3P	374666	0.491620112	1.53E-57	0.004147738
PILRB	29990	0.463687151	1.53E-51	0.007011653
CTSH	1512	0.48603352	2.54E-56	0.004690895
SAT1	6303	0.553072626	1.36E-71	0.001431957
JUNB	3726	0.452513966	3.30E-49	0.008641122
CD53	963	0.508379888	2.94E-61	0.00296267
PECAM1	5175	0.597765363	1.48E-82	0.000395023
IL10RA	3587	0.502793296	5.21E-60	0.00320956
RCSD1	92241	0.452513966	3.30E-49	0.008641122
ARHGDIB	397	0.452513966	3.30E-49	0.008641122
GIMAP5	55340	0.581005587	2.26E-78	0.000641912
GIMAP6	474344	0.474860335	6.52E-54	0.005629074
HLA-DMB	3109	0.597765363	1.48E-82	0.000395023
PTPRC	5788	0.502793296	5.21E-60	0.00320956
C10orf128	170371	0.502793296	5.21E-60	0.00320956
CMBL	134147	0.474860335	6.52E-54	0.005629074
HLA-DRB5	3127	0.558659218	6.26E-73	0.001234446
HLA-DPA1	3113	0.558659218	6.26E-73	0.001234446
ABCG1	9619	0.642458101	3.65E-94	0.000246889
GIMAP7	168537	0.480446927	4.11E-55	0.005184673
HLA-DQA1	3117	0.502793296	5.21E-60	0.00320956
TSHZ2	128553	0.463687151	1.53E-51	0.007011653
RGCC	28984	0.502793296	5.21E-60	0.00320956
CCR1	1230	0.502793296	5.21E-60	0.00320956
NPR3	4883	0.458100559	2.28E-50	0.007406676
RSAD2	91543	0.491620112	1.53E-57	0.004147738
GIMAP1	170575	0.474860335	6.52E-54	0.005629074
TNFSF10	8743	0.497206704	9.02E-59	0.003703338
AFTPH	54812	0.581005587	2.26E-78	0.000641912
NA	643187	0.458100559	2.28E-50	0.007406676
MALAT1	378938	0.497206704	9.02E-59	0.003703338
UBXN2A	165324	0.463687151	1.53E-51	0.007011653
PDE4C	5143	0.56424581	2.82E-74	0.000987557
GIMAP8	155038	0.474860335	6.52E-54	0.005629074
FYB	2533	0.547486034	2.87E-70	0.001530713
MS4A7	58475	0.525139665	4.62E-65	0.002024491
C5orf56	441108	0.458100559	2.28E-50	0.007406676
LOC400931	400931	0.474860335	6.52E-54	0.005629074
MLLT6	4302	0.664804469	3.21E-100	9.88E-05
CTSS	1520	0.48603352	2.54E-56	0.004690895

ZBTB20	26137	0.458100559	2.28E-50	0.007406676

Table s5: GO terms associated with the DNA replication / cell cycle expression module.

GO ID	p-value	Term
GO:0000280	7.52E-14	nuclear division
GO:0007067	7.52E-14	mitosis
GO:0048285	1.22E-13	organelle fission
GO:0000087	1.28E-13	M phase of mitotic cell cycle
GO:0022403	3.70E-13	cell cycle phase
GO:0000279	1.26E-12	M phase
GO:0000278	1.92E-12	mitotic cell cycle
GO:0022402	2.78E-12	cell cycle process
GO:0051301	3.40E-12	cell division
GO:0007049	3.88E-12	cell cycle
GO:0000070	6.02E-09	mitotic sister chromatid segregation
GO:0000819	7.13E-09	sister chromatid segregation
GO:0000226	2.29E-08	microtubule cytoskeleton organization
GO:0006996	4.19E-08	organelle organization
GO:0007059	6.75E-08	chromosome segregation
GO:0007051	7.94E-08	spindle organization
GO:0051276	8.06E-08	chromosome organization
GO:0000075	1.92E-07	cell cycle checkpoint
GO:0051656	3.08E-07	establishment of organelle localization
GO:0050000	4.99E-07	chromosome localization
GO:0051303	4.99E-07	establishment of chromosome localization
GO:0051726	9.53E-07	regulation of cell cycle
GO:0007017	1.09E-06	microtubule-based process
GO:0007093	1.63E-06	mitotic cell cycle checkpoint
GO:0051640	1.78E-06	organelle localization
GO:0006259	1.81E-06	DNA metabolic process
GO:0008608	3.22E-06	attachment of spindle microtubules to kinetochore
GO:0051313	3.22E-06	attachment of spindle microtubules to chromosome
GO:0007346	4.21E-06	regulation of mitotic cell cycle
GO:0040001	4.82E-06	establishment of mitotic spindle localization
GO:0006261	9.11E-06	DNA-dependent DNA replication
GO:0007080	9.42E-06	mitotic metaphase plate congression
GO:0051293	9.42E-06	establishment of spindle localization
GO:0051653	9.42E-06	spindle localization
GO:0007079	1.53E-05	mitotic chromosome movement towards spindle pole
GO:0051984	1.53E-05	positive regulation of chromosome segregation

GO:0051987	1.53E-05	positive regulation of attachment of spindle microtubules to kinetochore
GO:0051329	1.58E-05	interphase of mitotic cell cycle
GO:0051310	1.62E-05	metaphase plate congression
GO:0051325	2.26E-05	interphase
GO:0034453	2.57E-05	microtubule anchoring
GO:0010564	3.29E-05	regulation of cell cycle process
GO:0010638	3.35E-05	positive regulation of organelle organization
GO:0006260	3.41E-05	DNA replication
GO:0006189	4.59E-05	'de novo' IMP biosynthetic process
GO:0045842	4.59E-05	positive regulation of mitotic metaphase/anaphase transition
GO:0051305	4.59E-05	chromosome movement towards spindle pole
GO:0051988	4.59E-05	regulation of attachment of spindle microtubules to kinetochore
GO:0042770	5.20E-05	DNA damage response, signal transduction
GO:0070925	6.40E-05	organelle assembly
GO:0007052	7.38E-05	mitotic spindle organization
GO:0000077	8.44E-05	DNA damage checkpoint
GO:0045840	8.53E-05	positive regulation of mitosis
GO:0051225	8.53E-05	spindle assembly
GO:0051785	8.53E-05	positive regulation of nuclear division
GO:0006188	9.16E-05	IMP biosynthetic process
GO:0046040	9.16E-05	IMP metabolic process
GO:0031570	0.000102493	DNA integrity checkpoint
GO:0006270	0.000126262	DNA-dependent DNA replication initiation
GO:0045787	0.000138788	positive regulation of cell cycle
GO:0007095	0.000152304	mitotic cell cycle G2/M transition DNA damage checkpoint
GO:0034501	0.000152304	protein localization to kinetochore
GO:0043570	0.000152304	maintenance of DNA repeat elements
GO:0051096	0.000152304	positive regulation of helicase activity
GO:0071780	0.000152304	mitotic cell cycle G2/M transition checkpoint
GO:0007010	0.000158535	cytoskeleton organization
GO:0006974	0.000162218	response to DNA damage stimulus
GO:0002566	0.000227877	somatic diversification of immune receptors via somatic mutation
GO:0016446	0.000227877	somatic hypermutation of immunoglobulin genes
GO:0051383	0.000227877	kinetochore organization
GO:0000086	0.000242661	G2/M transition of mitotic cell cycle
GO:0031123	0.000242661	RNA 3'-end processing
GO:0000132	0.00031822	establishment of mitotic spindle orientation
GO:0051095	0.00031822	regulation of helicase activity

GO:0051294	0.00031822	establishment of spindle orientation
GO:0051297	0.00052015	centrosome organization
GO:0008340	0.000542761	determination of adult lifespan
GO:0010389	0.000542761	regulation of G2/M transition of mitotic cell cycle
GO:0045910	0.000542761	negative regulation of DNA recombination
GO:0031023	0.000559652	microtubule organizing center organization
GO:0090068	0.000644305	positive regulation of cell cycle process
GO:0016043	0.000661968	cellular component organization
GO:0090304	0.000751504	nucleic acid metabolic process
GO:0051716	0.000765834	cellular response to stimulus
GO:0006268	0.000825026	DNA unwinding involved in replication
GO:0051983	0.000987526	regulation of chromosome segregation
GO:0010259	0.001164124	multicellular organismal aging
GO:0031058	0.001164124	positive regulation of histone modification
GO:0071174	0.001164124	mitotic cell cycle spindle checkpoint
GO:0006139	0.001184437	nucleobase, nucleoside, nucleotide and nucleic acid metabolic process
GO:0033554	0.001264272	cellular response to stress
GO:0071103	0.001274869	DNA conformation change
GO:0034641	0.001471331	cellular nitrogen compound metabolic process
GO:0007088	0.001545082	regulation of mitosis
GO:0051783	0.001545082	regulation of nuclear division
GO:0032507	0.001787196	maintenance of protein location in cell
GO:0009127	0.00200931	purine nucleoside monophosphate biosynthetic process
GO:0009168	0.00200931	purine ribonucleoside monophosphate biosynthetic process
GO:0031577	0.00200931	spindle checkpoint
GO:0000082	0.002145096	G1/S transition of mitotic cell cycle
GO:0051130	0.002169458	positive regulation of cellular component organization
GO:0045185	0.002241011	maintenance of protein location
GO:0032392	0.002254764	DNA geometric change
GO:0032508	0.002254764	DNA duplex unwinding
GO:0006807	0.002269381	nitrogen compound metabolic process
GO:0051651	0.002440746	maintenance of location in cell
GO:0033043	0.002513612	regulation of organelle organization
GO:0016458	0.002651184	gene silencing
GO:0006298	0.002785911	mismatch repair
GO:0031572	0.002785911	G2/M transition DNA damage checkpoint
GO:0009126	0.003071393	purine nucleoside monophosphate metabolic process
GO:0009167	0.003071393	purine ribonucleoside monophosphate metabolic

		process
GO:0031056	0.003071393	regulation of histone modification
GO:0031124	0.003071393	mRNA 3'-end processing
GO:0000710	0.003955576	meiotic mismatch repair
GO:0003272	0.003955576	endocardial cushion formation
GO:0007100	0.003955576	mitotic centrosome separation
GO:0010610	0.003955576	regulation of mRNA stability involved in response to stress
GO:0021998	0.003955576	neural plate mediolateral regionalization
GO:0033129	0.003955576	positive regulation of histone phosphorylation
GO:0043146	0.003955576	spindle stabilization
GO:0043148	0.003955576	mitotic spindle stabilization
GO:0046680	0.003955576	response to DDT
GO:0048338	0.003955576	mesoderm structural organization
GO:0048352	0.003955576	paraxial mesoderm structural organization
GO:0060623	0.003955576	regulation of chromosome condensation
GO:0071281	0.003955576	cellular response to iron ion
GO:0071283	0.003955576	cellular response to iron(III) ion
GO:0002204	0.004006215	somatic recombination of immunoglobulin genes involved in immune response
GO:0002208	0.004006215	somatic diversification of immunoglobulins involved in immune response
GO:0007091	0.004006215	mitotic metaphase/anaphase transition
GO:0009156	0.004006215	ribonucleoside monophosphate biosynthetic process
GO:0030010	0.004006215	establishment of cell polarity
GO:0030071	0.004006215	regulation of mitotic metaphase/anaphase transition
GO:0031576	0.004006215	G2/M transition checkpoint
GO:0045190	0.004006215	isotype switching
GO:0010605	0.004216709	negative regulation of macromolecule metabolic process
GO:0008283	0.004296653	cell proliferation
GO:0002381	0.004343602	immunoglobulin production involved in immunoglobulin mediated immune response
GO:0006342	0.004693708	chromatin silencing
GO:0030261	0.004693708	chromosome condensation
GO:0051129	0.004995788	negative regulation of cellular component organization
GO:0009161	0.005431668	ribonucleoside monophosphate metabolic process
GO:0016447	0.005431668	somatic recombination of immunoglobulin gene segments

GO:0000018	0.005819321	regulation of DNA recombination
GO:0045814	0.005819321	negative regulation of gene expression, epigenetic
GO:0040029	0.005896798	regulation of gene expression, epigenetic
GO:0006281	0.006387647	DNA repair
GO:0009892	0.006597795	negative regulation of metabolic process
GO:0010639	0.006626223	negative regulation of organelle organization
GO:0016445	0.006631468	somatic diversification of immunoglobulins
GO:0008630	0.007492078	DNA damage response, signal transduction resulting in induction of apoptosis
GO:0000236	0.007895805	mitotic prometaphase
GO:0003203	0.007895805	endocardial cushion morphogenesis
GO:0009082	0.007895805	branched chain family amino acid biosynthetic process
GO:0010041	0.007895805	response to iron(III) ion
GO:0010424	0.007895805	DNA methylation on cytosine within a CG sequence
GO:0032776	0.007895805	DNA methylation on cytosine
GO:0033127	0.007895805	regulation of histone phosphorylation
GO:0048369	0.007895805	lateral mesoderm morphogenesis
GO:0048370	0.007895805	lateral mesoderm formation
GO:0048371	0.007895805	lateral mesodermal cell differentiation
GO:0048372	0.007895805	lateral mesodermal cell fate commitment
GO:0048377	0.007895805	lateral mesodermal cell fate specification
GO:0048378	0.007895805	regulation of lateral mesodermal cell fate specification
GO:0048382	0.007895805	mesendoderm development
GO:0051571	0.007895805	positive regulation of histone H3-K4 methylation
GO:0060897	0.007895805	neural plate regionalization
GO:0070562	0.007895805	regulation of vitamin D receptor signaling pathway
GO:0090307	0.007895805	spindle assembly involved in mitosis
GO:0032269	0.008382756	negative regulation of cellular protein metabolic process
GO:0002562	0.008872146	somatic diversification of immune receptors via germline recombination within a single locus
GO:0016444	0.008872146	somatic cell DNA recombination
GO:0048477	0.008872146	oogenesis
GO:0051235	0.009127171	maintenance of location
GO:0050767	0.009727988	regulation of neurogenesis
GO:0002200	0.009850495	somatic diversification of immune receptors
GO:0048863	0.010356874	stem cell differentiation
GO:0051248	0.010368518	negative regulation of protein metabolic process

GO:0006344	0.011820745	maintenance of chromatin silencing
GO:0010586	0.011820745	miRNA metabolic process
GO:0010587	0.011820745	miRNA catabolic process
GO:0031442	0.011820745	positive regulation of mRNA 3'-end processing
GO:0046499	0.011820745	S-adenosylmethioninamine metabolic process
GO:0048368	0.011820745	lateral mesoderm development
GO:0050685	0.011820745	positive regulation of mRNA processing
GO:0051299	0.011820745	centrosome separation
GO:0051573	0.011820745	negative regulation of histone H3-K9 methylation
GO:0060896	0.011820745	neural plate pattern specification
GO:0060914	0.011820745	heart formation
GO:0070507	0.011943695	regulation of microtubule cytoskeleton organization
GO:0031324	0.012021243	negative regulation of cellular metabolic process
GO:0006310	0.012383973	DNA recombination
GO:0033044	0.012494885	regulation of chromosome organization
GO:0051960	0.013012966	regulation of nervous system development
GO:0051053	0.013630083	negative regulation of DNA metabolic process
GO:0002377	0.015413557	immunoglobulin production
GO:0000089	0.015730456	mitotic metaphase
GO:0000281	0.015730456	cytokinesis after mitosis
GO:0001880	0.015730456	Mullerian duct regression
GO:0006269	0.015730456	DNA replication, synthesis of RNA primer
GO:0006346	0.015730456	methylation-dependent chromatin silencing
GO:0031062	0.015730456	positive regulation of histone methylation
GO:0031440	0.015730456	regulation of mRNA 3'-end processing
GO:0042661	0.015730456	regulation of mesodermal cell fate specification
GO:0045347	0.015730456	negative regulation of MHC class II biosynthetic process
GO:0051570	0.015730456	regulation of histone H3-K9 methylation
GO:0060218	0.015730456	hemopoietic stem cell differentiation
GO:0060236	0.015730456	regulation of mitotic spindle organization
GO:0070561	0.015730456	vitamin D receptor signaling pathway
GO:0072132	0.015730456	mesenchyme morphogenesis
GO:0032886	0.016029199	regulation of microtubule-based process
GO:0051495	0.017291676	positive regulation of cytoskeleton organization
GO:0040007	0.017363157	growth
GO:0042493	0.017388016	response to drug
GO:0031400	0.01786688	negative regulation of protein modification process
GO:0008629	0.017938333	induction of apoptosis by intracellular signals
GO:0060284	0.019513871	regulation of cell development
GO:0009628	0.01952189	response to abiotic stimulus

GO:0003197	0.019624993	endocardial cushion development
GO:0007501	0.019624993	mesodermal cell fate specification
GO:0010870	0.019624993	positive regulation of receptor biosynthetic process
GO:0030916	0.019624993	otic vesicle formation
GO:0031061	0.019624993	negative regulation of histone methylation
GO:0031573	0.019624993	intra-S DNA damage checkpoint
GO:0051382	0.019624993	kinetochore assembly
GO:0051569	0.019624993	regulation of histone H3-K4 methylation
GO:0070934	0.019624993	CRD-mediated mRNA stabilization
GO:0071305	0.019624993	cellular response to vitamin D
GO:0071398	0.019624993	cellular response to fatty acid
GO:0071453	0.019624993	cellular response to oxygen levels
GO:0071456	0.019624993	cellular response to hypoxia
GO:0071599	0.019624993	otic vesicle development
GO:0071600	0.019624993	otic vesicle morphogenesis
GO:0090224	0.019624993	regulation of spindle organization
GO:0007163	0.019938926	establishment or maintenance of cell polarity
GO:0014070	0.021040728	response to organic cyclic substance
GO:0009987	0.022113253	cellular process
GO:0044260	0.022685343	cellular macromolecule metabolic process
GO:0032268	0.022850588	regulation of cellular protein metabolic process
GO:0006398	0.023504417	histone mRNA 3'-end processing
GO:0031054	0.023504417	pre-microRNA processing
GO:0033762	0.023504417	response to glucagon stimulus
GO:0046498	0.023504417	S-adenosylhomocysteine metabolic process
GO:0051567	0.023504417	histone H3-K9 methylation
GO:0060033	0.023504417	anatomical structure regression
GO:0000079	0.024205165	regulation of cyclin-dependent protein kinase activity
GO:0009411	0.024205165	response to UV
GO:0031323	0.024229028	regulation of cellular metabolic process
GO:0016570	0.025724865	histone modification
GO:0002440	0.026466249	production of molecular mediator of immune response
GO:0006302	0.026466249	double-strand break repair
GO:0031145	0.026466249	anaphase-promoting complex-dependent proteasomal ubiquitin-dependent protein catabolic process
GO:0016569	0.026555857	covalent chromatin modification
GO:0016310	0.026882049	phosphorylation
GO:0034661	0.027368783	ncRNA catabolic process
GO:0051323	0.027368783	metaphase

GO:0060391	0.027368783	positive regulation of SMAD protein nuclear translocation
GO:0071396	0.027368783	cellular response to lipid
GO:0007292	0.028019516	female gamete generation
GO:0032270	0.028347257	positive regulation of cellular protein metabolic process
GO:0030900	0.029134926	forebrain development
GO:0010212	0.029608727	response to ionizing radiation
GO:0051439	0.029608727	regulation of ubiquitin-protein ligase activity involved in mitotic cell cycle
GO:0032880	0.030472794	regulation of protein localization
GO:0044237	0.03110202	cellular metabolic process
GO:0009113	0.031218149	purine base biosynthetic process
GO:0010224	0.031218149	response to UV-B
GO:0017085	0.031218149	response to insecticide
GO:0019047	0.031218149	provirus integration
GO:0030069	0.031218149	lysogeny
GO:0031060	0.031218149	regulation of histone methylation
GO:0034508	0.031218149	centromere complex assembly
GO:0048340	0.031218149	paraxial mesoderm morphogenesis
GO:0048532	0.031218149	anatomical structure arrangement
GO:0048853	0.031218149	forebrain morphogenesis
GO:0055015	0.031218149	ventricular cardiac muscle cell development
GO:0060045	0.031218149	positive regulation of cardiac muscle cell proliferation
GO:0060390	0.031218149	regulation of SMAD protein nuclear translocation
GO:0071407	0.031218149	cellular response to organic cyclic substance
GO:0016064	0.031233241	immunoglobulin mediated immune response
GO:0019724	0.032058539	B cell mediated immunity
GO:0007420	0.032187216	brain development
GO:0051247	0.033532315	positive regulation of protein metabolic process
GO:0009950	0.035052572	dorsal/ventral axis specification
GO:0010453	0.035052572	regulation of cell fate commitment
GO:0010470	0.035052572	regulation of gastrulation
GO:0016572	0.035052572	histone phosphorylation
GO:0031503	0.035052572	protein complex localization
GO:0033205	0.035052572	cell cycle cytokinesis
GO:0042659	0.035052572	regulation of cell fate specification
GO:0010243	0.036312306	response to organic nitrogen
GO:0051641	0.037096512	cellular localization
GO:0045786	0.037642407	negative regulation of cell cycle
GO:0051246	0.038616306	regulation of protein metabolic process
GO:0001710	0.03887211	mesodermal cell fate commitment

GO:0006301	0.03887211	postreplication repair
GO:0006303	0.03887211	double-strand break repair via nonhomologous end joining
GO:0006349	0.03887211	regulation of gene expression by genetic imprinting
GO:0006378	0.03887211	mRNA polyadenylation
GO:0010869	0.03887211	regulation of receptor biosynthetic process
GO:0031057	0.03887211	negative regulation of histone modification
GO:0043584	0.03887211	nose development
GO:0045346	0.03887211	regulation of MHC class II biosynthetic process
GO:0071241	0.03887211	cellular response to inorganic substance
GO:0071248	0.03887211	cellular response to metal ion
GO:0071514	0.03887211	genetic imprinting
GO:0046661	0.041686743	male sex differentiation
GO:0051438	0.041686743	regulation of ubiquitin-protein ligase activity
GO:0048015	0.042610059	phosphoinositide-mediated signaling
GO:0006379	0.042676819	mRNA cleavage
GO:0045342	0.042676819	MHC class II biosynthetic process
GO:0048333	0.042676819	mesodermal cell differentiation
GO:0055012	0.042676819	ventricular cardiac muscle cell differentiation
GO:0051128	0.043302372	regulation of cellular component organization
GO:0051340	0.044479666	regulation of ligase activity
GO:0048519	0.045547242	negative regulation of biological process
GO:0034645	0.045691844	cellular macromolecule biosynthetic process
GO:0007281	0.046379426	germ cell development
GO:0031099	0.046379426	regeneration
GO:0001556	0.046466754	oocyte maturation
GO:0002021	0.046466754	response to dietary excess
GO:0007076	0.046466754	mitotic chromosome condensation
GO:0007094	0.046466754	mitotic cell cycle spindle assembly checkpoint
GO:0009083	0.046466754	branched chain family amino acid catabolic process
GO:0010714	0.046466754	positive regulation of collagen metabolic process
GO:0032967	0.046466754	positive regulation of collagen biosynthetic process
GO:0046112	0.046466754	nucleobase biosynthetic process
GO:0051568	0.046466754	histone H3-K4 methylation
GO:0051094	0.046704657	positive regulation of developmental process
GO:0006950	0.047411532	response to stress

Table s6: GO terms associated with the RNA transcription / protein synthesis expression module.

GO ID	p-value	Term

GO:0006420	2.84E-05	arginyl-tRNA aminoacylation
GO:0018198	0.000197338	peptidyl-cysteine modification
GO:0009108	0.001505193	coenzyme biosynthetic process
GO:0008380	0.002033993	RNA splicing
GO:0006397	0.002458656	mRNA processing
GO:0022613	0.002766281	ribonucleoprotein complex biogenesis
GO:0007192	0.003118819	activation of adenylate cyclase activity by serotonin receptor signaling pathway
GO:0017014	0.003118819	protein amino acid nitrosylation
GO:0018119	0.003118819	peptidyl-cysteine S-nitrosylation
GO:0042660	0.003118819	positive regulation of cell fate specification
GO:0046294	0.003118819	formaldehyde catabolic process
GO:0048936	0.003118819	peripheral nervous system neuron axonogenesis
GO:0044281	0.003169195	small molecule metabolic process
GO:0051188	0.004581947	cofactor biosynthetic process
GO:0006520	0.005315717	cellular amino acid metabolic process
GO:0016071	0.005476853	mRNA metabolic process
GO:0000022	0.006228148	mitotic spindle elongation
GO:0000189	0.006228148	nuclear translocation of MAPK
GO:0019478	0.006228148	D-amino acid catabolic process
GO:0042699	0.006228148	follicle-stimulating hormone signaling pathway
GO:0046185	0.006228148	aldehyde catabolic process
GO:0046292	0.006228148	formaldehyde metabolic process
GO:0051231	0.006228148	spindle elongation
GO:0060128	0.006228148	adrenocorticotropin hormone secreting cell differentiation
GO:0060591	0.006228148	chondroblast differentiation
GO:0009987	0.006259244	cellular process
GO:0006396	0.00728534	RNA processing
GO:0006446	0.007904176	regulation of translational initiation
GO:0017157	0.008264316	regulation of exocytosis
GO:0006418	0.008631734	tRNA aminoacylation for protein translation
GO:0043038	0.008631734	amino acid activation
GO:0043039	0.008631734	tRNA aminoacylation
GO:0019752	0.009318116	carboxylic acid metabolic process
GO:0043436	0.009318116	oxoacid metabolic process
GO:0014889	0.009328015	muscle atrophy
GO:0017182	0.009328015	peptidyl-diphthamide metabolic process
GO:0017183	0.009328015	peptidyl-diphthamide biosynthetic process from peptidyl-histidine
GO:0018125	0.009328015	peptidyl-cysteine methylation

GO:0046416	0.009328015	D-amino acid metabolic process
GO:0060129	0.009328015	thyroid-stimulating hormone-secreting cell differentiation
GO:0070935	0.009328015	3'-UTR-mediated mRNA stabilization
GO:0044282	0.009730879	small molecule catabolic process
GO:0006082	0.009845979	organic acid metabolic process
GO:0042180	0.010395066	cellular ketone metabolic process
GO:0006732	0.012350571	coenzyme metabolic process
GO:0048511	0.012350571	rhythmic process
GO:0007008	0.012418447	outer mitochondrial membrane organization
GO:0043922	0.012418447	negative regulation by host of viral transcription
GO:0048935	0.012418447	peripheral nervous system neuron development
GO:0051409	0.012418447	response to nitrosative stress
GO:0070096	0.012418447	mitochondrial outer membrane translocase complex assembly
GO:0006413	0.014514097	translational initiation
GO:0044106	0.014817902	cellular amine metabolic process
GO:0021534	0.015499473	cell proliferation in hindbrain
GO:0021924	0.015499473	cell proliferation in the external granule layer
GO:0021930	0.015499473	granule cell precursor proliferation
GO:0032057	0.015499473	negative regulation of translational initiation in response to stress
GO:0048934	0.015499473	peripheral nervous system neuron differentiation
GO:0006067	0.018571121	ethanol metabolic process
GO:0006069	0.018571121	ethanol oxidation
GO:0007210	0.018571121	serotonin receptor signaling pathway
GO:0032055	0.018571121	negative regulation of translation in response to stress
GO:0032897	0.018571121	negative regulation of viral transcription
GO:0034308	0.018571121	monohydric alcohol metabolic process
GO:0060644	0.018571121	mammary gland epithelial cell differentiation
GO:0009063	0.019515168	cellular amino acid catabolic process
GO:0043921	0.021633418	modulation by host of viral transcription
GO:0046668	0.021633418	regulation of retinal cell programmed cell death
GO:0051775	0.021633418	response to redox state
GO:0052312	0.021633418	modulation of transcription in other organism involved in symbiotic interaction
GO:0052472	0.021633418	modulation by host of symbiont

		transcription
GO:0022618	0.022249871	ribonucleoprotein complex assembly
GO:0010001	0.022814877	glial cell differentiation
GO:0051301	0.023268534	cell division
GO:0006519	0.02370024	cellular amino acid and derivative metabolic process
GO:0009396	0.024686392	folic acid and derivative biosynthetic process
GO:0009435	0.024686392	NAD biosynthetic process
GO:0018202	0.024686392	peptidyl-histidine modification
GO:0043558	0.024686392	regulation of translational initiation in response to stress
GO:0046653	0.024686392	tetrahydrofolate metabolic process
GO:0046666	0.024686392	retinal cell programmed cell death
GO:0060045	0.024686392	positive regulation of cardiac muscle cell proliferation
GO:0009310	0.025133766	amine catabolic process
GO:0042698	0.025728003	ovulation cycle
GO:0051186	0.026128322	cofactor metabolic process
GO:0034622	0.026162461	cellular macromolecular complex assembly
GO:0002042	0.027730071	cell migration involved in sprouting angiogenesis
GO:0010453	0.027730071	regulation of cell fate commitment
GO:0019359	0.027730071	nicotinamide nucleotide biosynthetic process
GO:0021936	0.027730071	regulation of granule cell precursor proliferation
GO:0021940	0.027730071	positive regulation of granule cell precursor proliferation
GO:0030815	0.027730071	negative regulation of cAMP metabolic process
GO:0030818	0.027730071	negative regulation of cAMP biosynthetic process
GO:0042659	0.027730071	regulation of cell fate specification
GO:0043555	0.027730071	regulation of translation in response to stress
GO:0007188	0.028161812	G-protein signaling, coupled to cAMP nucleotide second messenger
GO:0042063	0.03068472	gliogenesis
GO:0030800	0.030764483	negative regulation of cyclic nucleotide metabolic process
GO:0030803	0.030764483	negative regulation of cyclic nucleotide biosynthetic process
GO:0030809	0.030764483	negative regulation of nucleotide

		biosynthetic process
GO:0043537	0.030764483	negative regulation of blood vessel endothelial cell migration
GO:0006412	0.03284547	translation
GO:0007128	0.033789655	meiotic prophase I
GO:0021984	0.033789655	adenohypophysis development
GO:0032855	0.033789655	positive regulation of Rac GTPase activity
GO:0051324	0.033789655	prophase
GO:0051851	0.033789655	modification by host of symbiont morphology or physiology
GO:0034660	0.03423083	ncRNA metabolic process
GO:0045761	0.034630745	regulation of adenylate cyclase activity
GO:0009308	0.035832323	amine metabolic process
GO:0000377	0.035987987	RNA splicing, via transesterification reactions with bulged adenosine as nucleophile
GO:0000398	0.035987987	nuclear mRNA splicing, via spliceosome
GO:0031279	0.035987987	regulation of cyclase activity
GO:0051339	0.036674296	regulation of lyase activity
GO:0006086	0.036805614	acetyl-CoA biosynthetic process from pyruvate
GO:0009083	0.036805614	branched chain family amino acid catabolic process
GO:0010510	0.036805614	regulation of acetyl-CoA biosynthetic process from pyruvate
GO:0045980	0.036805614	negative regulation of nucleotide metabolic process
GO:0051046	0.03692867	regulation of secretion
GO:0019933	0.038062107	cAMP-mediated signaling
GO:0010608	0.038117727	posttranscriptional regulation of gene expression
GO:0018193	0.038921335	peptidyl-amino acid modification
GO:0043536	0.039812388	positive regulation of blood vessel endothelial cell migration
GO:0045947	0.039812388	negative regulation of translational initiation
GO:0046782	0.039812388	regulation of viral transcription
GO:0055021	0.039812388	regulation of cardiac muscle tissue growth
GO:0055024	0.039812388	regulation of cardiac muscle tissue development
GO:0060043	0.039812388	regulation of cardiac muscle cell proliferation
GO:0044237	0.040070335	cellular metabolic process
GO:0000375	0.042344467	RNA splicing, via transesterification reactions

GO:0006085	0.042810004	acetyl-CoA biosynthetic process
GO:0006700	0.042810004	C21-steroid hormone biosynthetic process
GO:0006760	0.042810004	folic acid and derivative metabolic process
GO:0051193	0.042810004	regulation of cofactor metabolic process
GO:0051196	0.042810004	regulation of coenzyme metabolic process
GO:0034621	0.043195956	cellular macromolecular complex subunit organization
GO:0030817	0.045295615	regulation of cAMP biosynthetic process
GO:0014003	0.04579849	oligodendrocyte development
GO:0017158	0.04579849	regulation of calcium ion-dependent exocytosis
GO:0019080	0.04579849	viral genome expression
GO:0019083	0.04579849	viral transcription
GO:0019363	0.04579849	pyridine nucleotide biosynthetic process
GO:0060420	0.04579849	regulation of heart growth
GO:0006171	0.046799216	cAMP biosynthetic process
GO:0030814	0.046799216	regulation of cAMP metabolic process
GO:0051726	0.047999309	regulation of cell cycle
GO:0007018	0.048321133	microtubule-based movement
GO:0050709	0.048777871	negative regulation of protein secretion
GO:0051702	0.048777871	interaction with symbiont
GO:0006399	0.049088873	tRNA metabolic process
GO:0007187	0.04986109	G-protein signaling, coupled to cyclic nucleotide second messenger

Table s7: GO terms associated with the metabolism / hormone signaling expression module.

GO ID	p-value	Term
GO:0034660	0.001322169	ncRNA metabolic process
GO:0006399	0.001776558	tRNA metabolic process
GO:0042278	0.002085852	purine nucleoside metabolic process
GO:0046128	0.002085852	purine ribonucleoside metabolic process
GO:0006409	0.002129925	tRNA export from nucleus
GO:0009642	0.002129925	response to light intensity
GO:0015957	0.002129925	bis(5'-nucleosidyl) oligophosphate biosynthetic process
GO:0015960	0.002129925	diadenosine polyphosphate biosynthetic process
GO:0015965	0.002129925	diadenosine tetraphosphate metabolic process
GO:0015966	0.002129925	diadenosine tetraphosphate biosynthetic process
GO:0032289	0.002129925	myelin formation in the central nervous system
GO:0051031	0.002129925	tRNA transport
GO:0001942	0.003573516	hair follicle development
GO:0022404	0.003573516	molting cycle process

GO:0022405	0.003573516	hair cycle process
GO:0006418	0.00409276	tRNA aminoacylation for protein translation
GO:0042303	0.00409276	molting cycle
GO:0042633	0.00409276	hair cycle
GO:0043038	0.00409276	amino acid activation
GO:0043039	0.00409276	tRNA aminoacylation
GO:0006348	0.004255476	chromatin silencing at telomere
GO:0006426	0.004255476	glycyl-tRNA aminoacylation
GO:0006428	0.004255476	isoleucyl-tRNA aminoacylation
GO:0006481	0.004255476	C-terminal protein amino acid methylation
GO:0015942	0.004255476	formate metabolic process
GO:0018410	0.004255476	peptide or protein carboxyl-terminal blocking
GO:0042780	0.004255476	tRNA 3'-end processing
GO:0009119	0.004836233	ribonucleoside metabolic process
GO:0055086	0.005692612	nucleobase, nucleoside and nucleotide metabolic process
GO:0006475	0.00637666	internal protein amino acid acetylation
GO:0015956	0.00637666	bis(5'-nucleosidyl) oligophosphate metabolic process
GO:0015959	0.00637666	diadenosine polyphosphate metabolic process
GO:0022010	0.00637666	myelination in the central nervous system
GO:0032291	0.00637666	ensheathment of axons in the central nervous system
GO:0035315	0.00637666	hair cell differentiation
GO:0043628	0.00637666	ncRNA 3'-end processing
GO:0046499	0.00637666	S-adenosylmethioninamine metabolic process
GO:0051798	0.00637666	positive regulation of hair follicle development
GO:0009116	0.007645128	nucleoside metabolic process
GO:0007199	0.008493487	G-protein signaling, coupled to cGMP nucleotide second messenger
GO:0032276	0.008493487	regulation of gonadotropin secretion
GO:0032277	0.008493487	negative regulation of gonadotropin secretion
GO:0040016	0.008493487	embryonic cleavage
GO:0046880	0.008493487	regulation of follicle-stimulating hormone secretion
GO:0046882	0.008493487	negative regulation of follicle-stimulating hormone secretion
GO:0051797	0.008493487	regulation of hair follicle development
GO:0060218	0.008493487	hemopoietic stem cell differentiation
GO:0035264	0.009928836	multicellular organism growth
GO:0032288	0.010605965	myelin assembly
GO:0032926	0.010605965	negative regulation of activin receptor signaling pathway

GO:0042634	0.010605965	regulation of hair cycle
GO:0006283	0.012714102	transcription-coupled nucleotide-excision repair
GO:0032274	0.012714102	gonadotropin secretion
GO:0046498	0.012714102	S-adenosylhomocysteine metabolic process
GO:0046884	0.012714102	follicle-stimulating hormone secretion
GO:0070509	0.012714102	calcium ion import
GO:0070588	0.012714102	calcium ion transmembrane transport
GO:0000154	0.014817908	rRNA modification
GO:0030825	0.014817908	positive regulation of cGMP metabolic process
GO:0033683	0.014817908	nucleotide-excision repair, DNA incision
GO:0044237	0.016838242	cellular metabolic process
GO:0006465	0.01691739	signal peptide processing
GO:0009396	0.01691739	folic acid and derivative biosynthetic process
GO:0043249	0.01691739	erythrocyte maturation
GO:0043558	0.01691739	regulation of translational initiation in response to stress
GO:0045684	0.01691739	positive regulation of epidermis development
GO:0046653	0.01691739	tetrahydrofolate metabolic process
GO:0044281	0.017394375	small molecule metabolic process
GO:0009163	0.019012558	nucleoside biosynthetic process
GO:0019934	0.019012558	cGMP-mediated signaling
GO:0042451	0.019012558	purine nucleoside biosynthetic process
GO:0042455	0.019012558	ribonucleoside biosynthetic process
GO:0043555	0.019012558	regulation of translation in response to stress
GO:0044060	0.019012558	regulation of endocrine process
GO:0046129	0.019012558	purine ribonucleoside biosynthetic process
GO:0009650	0.021103419	UV protection
GO:0018196	0.021103419	peptidyl-asparagine modification
GO:0018279	0.021103419	protein amino acid N-linked glycosylation via asparagine
GO:0048820	0.021103419	hair follicle maturation
GO:0030823	0.023189983	regulation of cGMP metabolic process
GO:0060986	0.023189983	endocrine hormone secretion
GO:0007164	0.025272258	establishment of tissue polarity
GO:0006486	0.026347976	protein amino acid glycosylation
GO:0043413	0.026347976	macromolecule glycosylation
GO:0070085	0.026347976	glycosylation
GO:0032925	0.027350252	regulation of activin receptor signaling pathway
GO:0048821	0.027350252	erythrocyte development
GO:0044249	0.027781463	cellular biosynthetic process
GO:0044260	0.028257369	cellular macromolecule metabolic process
GO:0006760	0.029423975	folic acid and derivative metabolic process
GO:0034645	0.030926132	cellular macromolecule biosynthetic process

GO:0001502	0.031493433	cartilage condensation
GO:0014003	0.031493433	oligodendrocyte development
GO:0006730	0.032794344	one-carbon metabolic process
GO:0046483	0.032943656	heterocycle metabolic process
GO:0006725	0.033244252	cellular aromatic compound metabolic process
GO:0032924	0.033558636	activin receptor signaling pathway
GO:0009058	0.034305782	biosynthetic process
GO:0009416	0.03460864	response to light stimulus
GO:0002244	0.035619593	hemopoietic progenitor cell differentiation
GO:0043616	0.035619593	keratinocyte proliferation
GO:0071695	0.035619593	anatomical structure maturation
GO:0009059	0.035896956	macromolecule biosynthetic process
GO:0008152	0.036403368	metabolic process
GO:0010558	0.036475033	negative regulation of macromolecule biosynthetic process
GO:0031069	0.037676311	hair follicle morphogenesis
GO:0006519	0.038301916	cellular amino acid and derivative metabolic process
GO:0031327	0.040019133	negative regulation of cellular biosynthetic process
GO:0030968	0.041777065	endoplasmic reticulum unfolded protein response
GO:0034620	0.041777065	cellular response to unfolded protein
GO:0043009	0.041931225	chordate embryonic development
GO:0009890	0.042699542	negative regulation of biosynthetic process
GO:0009792	0.043082223	embryo development ending in birth or egg hatching
GO:0000718	0.043821118	nucleotide-excision repair, DNA damage removal
GO:0007223	0.043821118	Wnt receptor signaling pathway, calcium modulating pathway
GO:0045682	0.043821118	regulation of epidermis development
GO:0046068	0.043821118	cGMP metabolic process
GO:0009987	0.045108181	cellular process
GO:0009101	0.045768921	glycoprotein biosynthetic process
GO:0042558	0.045860967	pteridine and derivative metabolic process
GO:0006412	0.049386928	translation
GO:0045055	0.049928082	regulated secretory pathway
GO:0048730	0.049928082	epidermis morphogenesis

Table s8: GO terms associated with the signaling / cellular identity expression module.

GO ID	p-value	Term

GO:0006955	1.69E-08	immune response
GO:0002376	2.37E-08	immune system process
GO:0002504	4.25E-06	antigen processing and presentation of peptide or polysaccharide antigen via MHC class II
GO:0001910	2.04E-05	regulation of leukocyte mediated cytotoxicity
GO:0001911	3.22E-05	negative regulation of leukocyte mediated cytotoxicity
GO:0031341	3.34E-05	regulation of cell killing
GO:0031342	5.36E-05	negative regulation of cell killing
GO:0042492	5.36E-05	gamma-delta T cell differentiation
GO:0045586	5.36E-05	regulation of gamma-delta T cell differentiation
GO:0045588	5.36E-05	positive regulation of gamma-delta T cell differentiation
GO:0046643	5.36E-05	regulation of gamma-delta T cell activation
GO:0046645	5.36E-05	positive regulation of gamma-delta T cell activation
GO:0001909	6.18E-05	leukocyte mediated cytotoxicity
GO:0002704	0.00011219	negative regulation of leukocyte mediated immunity
GO:0002707	0.00011219	negative regulation of lymphocyte mediated immunity
GO:0002925	0.00011219	positive regulation of humoral immune response mediated by circulating immunoglobulin
GO:0033687	0.00011219	osteoblast proliferation
GO:0046629	0.00011219	gamma-delta T cell activation
GO:0002922	0.000149366	positive regulation of humoral immune response
GO:0002923	0.000149366	regulation of humoral immune response mediated by circulating immunoglobulin
GO:0002706	0.000215899	regulation of lymphocyte mediated immunity
GO:0019882	0.000271484	antigen processing and presentation
GO:0002714	0.000292106	positive regulation of B cell mediated immunity
GO:0002891	0.000292106	positive regulation of immunoglobulin mediated immune response
GO:0001906	0.000302434	cell killing
GO:0002703	0.00035299	regulation of leukocyte mediated immunity
GO:0002920	0.000413044	regulation of humoral immune response
GO:0065007	0.000531015	biological regulation

GO:0050789	0.000672523	regulation of biological process
GO:0002715	0.000715957	regulation of natural killer cell mediated immunity
GO:0042269	0.000715957	regulation of natural killer cell mediated cytotoxicity
GO:0001912	0.00080427	positive regulation of leukocyte mediated cytotoxicity
GO:0002698	0.00080427	negative regulation of immune effector process
GO:0050794	0.000941615	regulation of cellular process
GO:0050896	0.001113031	response to stimulus
GO:0031343	0.001207177	positive regulation of cell killing
GO:0046635	0.001207177	positive regulation of alpha-beta T cell activation
GO:0002683	0.001214137	negative regulation of immune system process
GO:0002712	0.001438112	regulation of B cell mediated immunity
GO:0002889	0.001438112	regulation of immunoglobulin mediated immune response
GO:0002252	0.001521832	immune effector process
GO:0002228	0.001560873	natural killer cell mediated immunity
GO:0042267	0.001560873	natural killer cell mediated cytotoxicity
GO:0002697	0.001840539	regulation of immune effector process
GO:0002824	0.001958061	positive regulation of adaptive immune response based on somatic recombination of immune receptors built from immunoglobulin superfamily domains
GO:0050777	0.001958061	negative regulation of immune response
GO:0002449	0.00205033	lymphocyte mediated immunity
GO:0002821	0.002100019	positive regulation of adaptive immune response
GO:0045582	0.002100019	positive regulation of T cell differentiation
GO:0002705	0.002246722	positive regulation of leukocyte mediated immunity
GO:0002708	0.002246722	positive regulation of lymphocyte mediated immunity
GO:0002158	0.002358132	osteoclast proliferation
GO:0002361	0.002358132	CD4-positive, CD25-positive, alpha-beta regulatory T cell differentiation
GO:0002370	0.002358132	natural killer cell cytokine production
GO:0002727	0.002358132	regulation of natural killer cell cytokine production
GO:0002729	0.002358132	positive regulation of natural killer cell cytokine production

GO:0009720	0.002358132	detection of hormone stimulus
GO:0009726	0.002358132	detection of endogenous stimulus
GO:0032829	0.002358132	regulation of CD4-positive, CD25-positive, alpha-beta regulatory T cell differentiation
GO:0032831	0.002358132	positive regulation of CD4-positive, CD25-positive, alpha-beta regulatory T cell differentiation
GO:0034436	0.002358132	glycoprotein transport
GO:0045838	0.002358132	positive regulation of membrane potential
GO:0050904	0.002358132	diapedesis
GO:0060448	0.002358132	dichotomous subdivision of terminal units involved in lung branching
GO:0045621	0.002398149	positive regulation of lymphocyte differentiation
GO:0046634	0.002398149	regulation of alpha-beta T cell activation
GO:0002455	0.003404688	humoral immune response mediated by circulating immunoglobulin
GO:0007204	0.003545142	elevation of cytosolic calcium ion concentration
GO:0002443	0.003699526	leukocyte mediated immunity
GO:0065008	0.004027722	regulation of biological quality
GO:0002700	0.004167465	regulation of production of molecular mediator of immune response
GO:0051480	0.004272108	cytosolic calcium ion homeostasis
GO:0001915	0.004710882	negative regulation of T cell mediated cytotoxicity
GO:0002716	0.004710882	negative regulation of natural killer cell mediated immunity
GO:0034314	0.004710882	Arp2/3 complex-mediated actin nucleation
GO:0045591	0.004710882	positive regulation of regulatory T cell differentiation
GO:0045953	0.004710882	negative regulation of natural killer cell mediated cytotoxicity
GO:0050855	0.004710882	regulation of B cell receptor signaling pathway
GO:0051607	0.004786756	defense response to virus
GO:0002699	0.005221786	positive regulation of immune effector process
GO:0060402	0.005221786	calcium ion transport into cytosol
GO:0046631	0.005445889	alpha-beta T cell activation
GO:0060401	0.005674356	cytosolic calcium ion transport
GO:0045580	0.005907169	regulation of T cell differentiation
GO:0002822	0.006385745	regulation of adaptive immune response based on somatic recombination of

		immune receptors built from immunoglobulin superfamily domains
GO:0032879	0.006415683	regulation of localization
GO:0002819	0.006631468	regulation of adaptive immune response
GO:0002032	0.007058262	desensitization of G-protein coupled receptor protein signaling pathway by arrestin
GO:0002378	0.007058262	immunoglobulin biosynthetic process
GO:0045542	0.007058262	positive regulation of cholesterol biosynthetic process
GO:0045589	0.007058262	regulation of regulatory T cell differentiation
GO:0045896	0.007058262	regulation of transcription, mitotic
GO:0045897	0.007058262	positive regulation of transcription, mitotic
GO:0046021	0.007058262	regulation of transcription from RNA polymerase II promoter, mitotic
GO:0046022	0.007058262	positive regulation of transcription from RNA polymerase II promoter, mitotic
GO:0006917	0.00726145	induction of apoptosis
GO:0012502	0.007337971	induction of programmed cell death
GO:0045619	0.007923631	regulation of lymphocyte differentiation
GO:0048878	0.008359535	chemical homeostasis
GO:0045088	0.009319878	regulation of innate immune response
GO:0002710	0.009400284	negative regulation of T cell mediated immunity
GO:0033688	0.009400284	regulation of osteoblast proliferation
GO:0034113	0.009400284	heterotypic cell-cell adhesion
GO:0090205	0.009400284	positive regulation of cholesterol metabolic process
GO:0002440	0.009906968	production of molecular mediator of immune response
GO:0002521	0.010351705	leukocyte differentiation
GO:0006874	0.010942755	cellular calcium ion homeostasis
GO:2000021	0.011129305	regulation of ion homeostasis
GO:0045010	0.011736959	actin nucleation
GO:0045019	0.011736959	negative regulation of nitric oxide biosynthetic process
GO:0045066	0.011736959	regulatory T cell differentiation
GO:0050857	0.011736959	positive regulation of antigen receptor-mediated signaling pathway
GO:0016064	0.011764243	immunoglobulin mediated immune response
GO:0055074	0.012023642	calcium ion homeostasis
GO:0019724	0.012087588	B cell mediated immunity

GO:0006875	0.012668084	cellular metal ion homeostasis
GO:0050870	0.013762313	positive regulation of T cell activation
GO:0001916	0.0140683	positive regulation of T cell mediated cytotoxicity
GO:0007171	0.0140683	activation of transmembrane receptor protein tyrosine kinase activity
GO:0010887	0.0140683	negative regulation of cholesterol storage
GO:0031953	0.0140683	negative regulation of protein amino acid autophosphorylation
GO:0032366	0.0140683	intracellular sterol transport
GO:0032367	0.0140683	intracellular cholesterol transport
GO:0045059	0.0140683	positive thymic T cell selection
GO:0048304	0.0140683	positive regulation of isotype switching to IgG isotypes
GO:0055091	0.0140683	phospholipid homeostasis
GO:0060136	0.0140683	embryonic process involved in female pregnancy
GO:0055065	0.014365205	metal ion homeostasis
GO:0002573	0.015170568	myeloid leukocyte differentiation
GO:0010740	0.015260172	positive regulation of intracellular protein kinase cascade
GO:0006959	0.015531987	humoral immune response
GO:0001914	0.016394319	regulation of T cell mediated cytotoxicity
GO:0002031	0.016394319	G-protein coupled receptor internalization
GO:0006198	0.016394319	cAMP catabolic process
GO:0032689	0.016394319	negative regulation of interferon-gamma production
GO:0045060	0.016394319	negative thymic T cell selection
GO:0045824	0.016394319	negative regulation of innate immune response
GO:0060600	0.016394319	dichotomous subdivision of an epithelial terminal unit
GO:0035556	0.01664198	intracellular signal transduction
GO:0019221	0.017777681	cytokine-mediated signaling pathway
GO:0023036	0.017777681	initiation of signal transduction
GO:0023038	0.017777681	signal initiation by diffusible mediator
GO:0023049	0.017777681	signal initiation by protein/peptide mediator
GO:0043410	0.017777681	positive regulation of MAPKKK cascade
GO:0010872	0.018715026	regulation of cholesterol esterification
GO:0032365	0.018715026	intracellular lipid transport
GO:0043011	0.018715026	myeloid dendritic cell differentiation
GO:0043368	0.018715026	positive T cell selection
GO:0043383	0.018715026	negative T cell selection

GO:0046641	0.018715026	positive regulation of alpha-beta T cell proliferation
GO:0048302	0.018715026	regulation of isotype switching to IgG isotypes
GO:0030005	0.018740757	cellular di-, tri-valent inorganic cation homeostasis
GO:0006952	0.019140405	defense response
GO:0050776	0.01936046	regulation of immune response
GO:0030217	0.020972695	T cell differentiation
GO:0002820	0.021030435	negative regulation of adaptive immune response
GO:0002823	0.021030435	negative regulation of adaptive immune response based on somatic recombination of immune receptors built from immunoglobulin superfamily domains
GO:0009214	0.021030435	cyclic nucleotide catabolic process
GO:0010893	0.021030435	positive regulation of steroid biosynthetic process
GO:0042987	0.021030435	amyloid precursor protein catabolic process
GO:0043372	0.021030435	positive regulation of CD4-positive, alpha beta T cell differentiation
GO:0045540	0.021030435	regulation of cholesterol biosynthetic process
GO:0045830	0.021030435	positive regulation of isotype switching
GO:0046902	0.021030435	regulation of mitochondrial membrane permeability
GO:0048291	0.021030435	isotype switching to IgG isotypes
GO:0045597	0.021730044	positive regulation of cell differentiation
GO:0055066	0.021730044	di-, tri-valent inorganic cation homeostasis
GO:0043065	0.021732802	positive regulation of apoptosis
GO:0043068	0.022200664	positive regulation of programmed cell death
GO:0007165	0.022734777	signal transduction
GO:0010942	0.022994253	positive regulation of cell death
GO:0001913	0.023340555	T cell mediated cytotoxicity
GO:0030146	0.023340555	diuresis
GO:0033700	0.023340555	phospholipid efflux
GO:0034374	0.023340555	low-density lipoprotein particle remodeling
GO:0045911	0.023340555	positive regulation of DNA recombination
GO:0030003	0.024489935	cellular cation homeostasis
GO:0051251	0.024830961	positive regulation of lymphocyte activation
GO:0001773	0.0256454	myeloid dendritic cell activation
GO:0002029	0.0256454	desensitization of G-protein coupled

		receptor protein signaling pathway
GO:0002720	0.0256454	positive regulation of cytokine production involved in immune response
GO:0010634	0.0256454	positive regulation of epithelial cell migration
GO:0022401	0.0256454	negative adaptation of signaling pathway
GO:0023058	0.0256454	adaptation of signaling pathway
GO:0031648	0.0256454	protein destabilization
GO:0031952	0.0256454	regulation of protein amino acid autophosphorylation
GO:0034433	0.0256454	steroid esterification
GO:0034434	0.0256454	sterol esterification
GO:0034435	0.0256454	cholesterol esterification
GO:0045061	0.0256454	thymic T cell selection
GO:0045123	0.0256454	cellular extravasation
GO:0050732	0.0256454	negative regulation of peptidyl-tyrosine phosphorylation
GO:0050853	0.0256454	B cell receptor signaling pathway
GO:0046907	0.026085117	intracellular transport
GO:0009967	0.026679788	positive regulation of signal transduction
GO:0051235	0.027090738	maintenance of location
GO:0023056	0.027940783	positive regulation of signaling process
GO:0001960	0.027944981	negative regulation of cytokine-mediated signaling pathway
GO:0002711	0.027944981	positive regulation of T cell mediated immunity
GO:0003091	0.027944981	renal water homeostasis
GO:0009125	0.027944981	nucleoside monophosphate catabolic process
GO:0010885	0.027944981	regulation of cholesterol storage
GO:0046640	0.027944981	regulation of alpha-beta T cell proliferation
GO:0046697	0.027944981	decidualization
GO:0090181	0.027944981	regulation of cholesterol metabolic process
GO:0002460	0.02943091	adaptive immune response based on somatic recombination of immune receptors built from immunoglobulin superfamily domains
GO:0002696	0.02990841	positive regulation of leukocyte activation
GO:0007187	0.02990841	G-protein signaling, coupled to cyclic nucleotide second messenger
GO:0001829	0.030239309	trophectodermal cell differentiation
GO:0006607	0.030239309	NLS-bearing substrate import into nucleus
GO:0010745	0.030239309	negative regulation of macrophage derived foam cell differentiation

GO:0010878	0.030239309	cholesterol storage
GO:0043370	0.030239309	regulation of CD4-positive, alpha beta T cell differentiation
GO:0045191	0.030239309	regulation of isotype switching
GO:0045577	0.030239309	regulation of B cell differentiation
GO:0050891	0.030239309	multicellular organismal water homeostasis
GO:0002250	0.030389025	adaptive immune response
GO:0050863	0.030872742	regulation of T cell activation
GO:0048585	0.03234233	negative regulation of response to stimulus
GO:0050867	0.03234233	positive regulation of cell activation
GO:0002717	0.032528396	positive regulation of natural killer cell mediated immunity
GO:0010631	0.032528396	epithelial cell migration
GO:0010632	0.032528396	regulation of epithelial cell migration
GO:0010888	0.032528396	negative regulation of lipid storage
GO:0034375	0.032528396	high-density lipoprotein particle remodeling
GO:0042147	0.032528396	retrograde transport, endosome to Golgi
GO:0042994	0.032528396	cytoplasmic sequestering of transcription factor
GO:0045954	0.032528396	positive regulation of natural killer cell mediated cytotoxicity
GO:0050854	0.032528396	regulation of antigen receptor-mediated signaling pathway
GO:0050995	0.032528396	negative regulation of lipid catabolic process
GO:0060716	0.032528396	labyrinthine layer blood vessel development
GO:0090132	0.032528396	epithelium migration
GO:0055080	0.032742446	cation homeostasis
GO:0046058	0.032838285	cAMP metabolic process
GO:0001893	0.034812254	maternal placenta development
GO:0002702	0.034812254	positive regulation of production of molecular mediator of immune response
GO:0032091	0.034812254	negative regulation of protein binding
GO:0046633	0.034812254	alpha-beta T cell proliferation
GO:0070661	0.034852141	leukocyte proliferation
GO:0019216	0.036393627	regulation of lipid metabolic process
GO:0051649	0.036897528	establishment of localization in cell
GO:0002709	0.037090894	regulation of T cell mediated immunity
GO:0042982	0.037090894	amyloid precursor protein metabolic process
GO:0046676	0.037090894	negative regulation of insulin secretion

GO:0051208	0.037090894	sequestering of calcium ion
GO:0090130	0.037090894	tissue migration
GO:0030097	0.03765206	hemopoiesis
GO:0030098	0.03796129	lymphocyte differentiation
GO:0045595	0.038541331	regulation of cell differentiation
GO:0032844	0.039020736	regulation of homeostatic process
GO:0043691	0.039364327	reverse cholesterol transport
GO:0045058	0.039364327	T cell selection
GO:0045940	0.039364327	positive regulation of steroid metabolic process
GO:0090278	0.039364327	negative regulation of peptide hormone secretion
GO:0006606	0.039554713	protein import into nucleus
GO:0019935	0.0406311	cyclic-nucleotide-mediated signaling
GO:0042592	0.040906208	homeostatic process
GO:0010627	0.041021136	regulation of intracellular protein kinase cascade
GO:0051170	0.041173479	nuclear import
GO:0002792	0.041632566	negative regulation of peptide secretion
GO:0006516	0.041632566	glycoprotein catabolic process
GO:0030104	0.041632566	water homeostasis
GO:0030838	0.041632566	positive regulation of actin filament polymerization
GO:0046638	0.041632566	positive regulation of alpha-beta T cell differentiation
GO:0051220	0.041632566	cytoplasmic sequestering of protein
GO:0051412	0.041632566	response to corticosterone stimulus
GO:0060441	0.041632566	epithelial tube branching involved in lung morphogenesis
GO:0019222	0.042224827	regulation of metabolic process
GO:0031400	0.042817175	negative regulation of protein modification process
GO:0048534	0.043888965	hemopoietic or lymphoid organ development
GO:0001825	0.043895621	blastocyst formation
GO:0002718	0.043895621	regulation of cytokine production involved in immune response
GO:0042992	0.043895621	negative regulation of transcription factor import into nucleus
GO:0043029	0.043895621	T cell homeostasis
GO:0060674	0.043895621	placenta blood vessel development
GO:0009187	0.044485396	cyclic nucleotide metabolic process
GO:0043367	0.046153505	CD4-positive, alpha beta T cell differentiation

GO:0006810	0.04615684	transport
GO:0007243	0.046177765	intracellular protein kinase cascade
GO:0023014	0.046177765	signal transmission via phosphorylation event
GO:0051094	0.046521539	positive regulation of developmental process
GO:0042308	0.048406228	negative regulation of protein import into nucleus
GO:0045744	0.048406228	negative regulation of G-protein coupled receptor protein signaling pathway
GO:0015031	0.048818151	protein transport
GO:0034504	0.049050825	protein localization in nucleus
GO:0051707	0.049921612	response to other organism

GEO Samples Included in the Concordia Database

GSM175794, GSM170979, GSM175795, GSM46884, GSM175796, GSM175797, GSM170978, GSM175790, GSM175791, GSM46888, GSM175792, GSM117730, GSM203686, GSM402327, GSM175793, GSM175798, GSM353935, GSM175799, GSM159011, GSM352110, GSM353933, GSM203696, GSM318104, GSM402317, GSM117720, GSM203699, GSM46878, GSM159001, GSM117710, GSM402307, GSM353915, GSM159031, GSM152689, GSM318124, GSM117700, GSM152681, GSM379868, GSM117701, GSM46898, GSM352123, GSM353925, GSM159021, GSM152699, GSM318114, GSM379858, GSM363401, GSM260997, GSM194307, GSM363406, GSM363403, GSM117770, GSM117772, GSM187610, GSM261007, GSM187611, GSM350298, GSM318144, GSM187616, GSM194309, GSM187617, GSM194308, GSM187618, GSM187619, GSM187612, GSM187613, GSM187614, GSM152669, GSM187615, GSM194313, GSM194314, GSM194311, GSM353905, GSM194312, GSM199397, GSM117763, GSM194310, GSM76489, GSM117761, GSM261017, GSM117756, GSM187621, GSM67186, GSM187622, GSM117755, GSM152670, GSM187620, GSM318134, GSM350288, GSM187629, GSM152679, GSM187627, GSM187628, GSM187625, GSM187626, GSM187623, GSM187624, GSM175777, GSM175776, GSM260977, GSM175779, GSM175778, GSM76499, GSM117751, GSM175775, GSM187630, GSM337197, GSM152649, GSM337199, GSM337198, GSM385721, GSM363411, GSM175789, GSM363412, GSM175788, GSM260987, GSM175787, GSM325807, GSM175782, GSM175781, GSM117741, GSM175780, GSM175786, GSM363415, GSM175785, GSM175784, GSM175783, GSM280370, GSM152659, GSM361954, GSM391367, GSM211122, GSM280847, GSM371106, GSM148611, GSM148610, GSM211132, GSM325817, GSM85486, GSM325812, GSM361964, GSM391357, GSM280837, GSM325827, GSM148605, GSM211142,

GSM148606, GSM148607, GSM148608, GSM148609, GSM85496, GSM260967, GSM279060, GSM279061, GSM279062, GSM279063, GSM279064, GSM279065, GSM211102, GSM46824, GSM348321, GSM325837, GSM46828, GSM211112, GSM151998, GSM151999, GSM151996, GSM151997, GSM151994, GSM151995, GSM151992, GSM151993, GSM151990, GSM46818, GSM151991, GSM46817, GSM85476, GSM238798, GSM201248, GSM238799, GSM201249, GSM201246, GSM201247, GSM201244, GSM201245, GSM270842, GSM270843, GSM270844, GSM270840, GSM261088, GSM231885, GSM270841, GSM231886, GSM46848, GSM151980, GSM261092, GSM151982, GSM261091, GSM151981, GSM151984, GSM201254, GSM151983, GSM201253, GSM151986, GSM201252, GSM151985, GSM201251, GSM151988, GSM201250, GSM151987, GSM151989, GSM201259, GSM231899, GSM201255, GSM201256, GSM201257, GSM201258, GSM270834, GSM261096, GSM261099, GSM231896, GSM231897, GSM46838, GSM270839, GSM270838, GSM151971, GSM270837, GSM151970, GSM270836, GSM270835, GSM151975, GSM201263, GSM151974, GSM201262, GSM151973, GSM201265, GSM151972, GSM201264, GSM301697, GSM151979, GSM151978, GSM151977, GSM201261, GSM46833, GSM151976, GSM201260, GSM151969, GSM151966, GSM151965, GSM151968, GSM46868, GSM151967, GSM151962, GSM201232, GSM201231, GSM151964, GSM201230, GSM151963, GSM201233, GSM201234, GSM201235, GSM201236, GSM201237, GSM385383, GSM201238, GSM201239, GSM231876, GSM231874, GSM46858, GSM238795, GSM238794, GSM238797, GSM238796, GSM238791, GSM201241, GSM238790, GSM201240, GSM46850, GSM238793, GSM201243, GSM238792, GSM279753, GSM173679, GSM325787, GSM53033, GSM386413, GSM60985, GSM173684, GSM317736, GSM279743, GSM173685, GSM173682, GSM173683, GSM306190, GSM173680, GSM173681, GSM211092, GSM317739, GSM80602, GSM80601, GSM80600, GSM173688, GSM270809, GSM173689, GSM173686, GSM173687, GSM60972, GSM386403, GSM316693, GSM238875, GSM238877, GSM238870, GSM211082, GSM238873, GSM280897, GSM279774, GSM238874, GSM238871, GSM238872, GSM351404, GSM238867, GSM238865, GSM238864, GSM316683, GSM238868, GSM211072, GSM238860, GSM238861, GSM199307, GSM238862, GSM279763, GSM238863, GSM66937, GSM325797, GSM360316, GSM238854, GSM238856, GSM238855, GSM238858, GSM238857, GSM316673, GSM80632, GSM80633, GSM80634, GSM80635, GSM80630, GSM80631, GSM340514, GSM372286, GSM238851, GSM280877, GSM372289, GSM372288, GSM372287, GSM238848, GSM401152, GSM238846, GSM238847, GSM372292, GSM238844, GSM401156, GSM372293, GSM238845, GSM372290, GSM238842, GSM372291, GSM238843, GSM80629, GSM386453, GSM80626, GSM80625, GSM360329,

GSM80628, GSM80627, GSM80645, GSM80646, GSM80643, GSM75017, GSM80644, GSM80641, GSM340504, GSM80642, GSM80640, GSM372295, GSM372294, GSM280887, GSM372297, GSM238841, GSM372296, GSM279784, GSM238840, GSM372299, GSM372298, GSM401162, GSM238835, GSM238837, GSM238838, GSM401165, GSM279794, GSM238834, GSM386443, GSM80639, GSM238839, GSM80638, GSM80637, GSM80636, GSM80610, GSM176306, GSM80611, GSM203716, GSM80612, GSM176304, GSM80613, GSM176305, GSM176302, GSM176303, GSM352580, GSM176300, GSM176301, GSM238822, GSM280857, GSM238823, GSM238820, GSM401132, GSM238821, GSM238826, GSM238827, GSM238824, GSM238825, GSM80604, GSM80603, GSM60960, GSM80606, GSM80605, GSM386433, GSM80608, GSM80607, GSM80609, GSM176319, GSM179951, GSM80620, GSM179950, GSM80623, GSM176315, GSM80624, GSM176316, GSM80621, GSM176317, GSM203706, GSM80622, GSM176318, GSM176312, GSM176313, GSM176310, GSM238810, GSM280867, GSM238811, GSM238812, GSM238813, GSM401142, GSM238815, GSM238816, GSM80617, GSM386423, GSM238817, GSM80616, GSM238818, GSM80615, GSM238819, GSM80614, GSM80619, GSM80618, GSM152759, GSM152757, GSM187702, GSM350248, GSM238807, GSM152755, GSM238806, GSM80669, GSM238809, GSM238808, GSM238803, GSM238802, GSM238805, GSM238804, GSM401112, GSM238801, GSM238800, GSM80671, GSM203732, GSM80670, GSM176321, GSM176320, GSM117680, GSM176323, GSM203736, GSM176322, GSM175840, GSM176325, GSM175841, GSM176324, GSM80679, GSM175842, GSM176327, GSM80678, GSM175843, GSM176326, GSM80677, GSM175844, GSM176329, GSM80676, GSM175845, GSM176328, GSM80675, GSM175846, GSM80674, GSM175847, GSM179940, GSM80673, GSM175848, GSM199357, GSM80672, GSM175849, GSM175839, GSM152749, GSM350258, GSM345187, GSM401122, GSM80680, GSM176332, GSM176331, GSM80682, GSM176330, GSM80681, GSM176336, GSM175830, GSM176335, GSM176334, GSM176333, GSM203726, GSM80688, GSM175833, GSM179930, GSM80687, GSM301707, GSM175834, GSM117690, GSM176339, GSM175831, GSM176338, GSM80689, GSM175832, GSM176337, GSM80684, GSM175837, GSM80683, GSM175838, GSM199367, GSM80686, GSM175835, GSM80685, GSM175836, GSM80649, GSM80647, GSM80648, GSM187722, GSM281019, GSM350268, GSM175860, GSM176345, GSM175861, GSM176344, GSM175862, GSM117660, GSM176347, GSM203756, GSM175863, GSM176346, GSM176341, GSM176340, GSM176343, GSM176342, GSM80653, GSM175868, GSM80652, GSM175869, GSM80651, GSM340534, GSM80650, GSM152739, GSM80657, GSM53093, GSM175864, GSM199377, GSM80656, GSM175865, GSM80655, GSM175866, GSM80654, GSM175867, GSM179920, GSM80658, GSM80659, GSM281009, GSM187712, GSM176360, GSM401102, GSM176361, GSM350278, GSM175851, GSM176358, GSM175852, GSM176357,

GSM203746, GSM176356, GSM175850, GSM117670, GSM176355, GSM176354, GSM176353, GSM80660, GSM176352, GSM179918, GSM80662, GSM368398, GSM175859, GSM152729, GSM80661, GSM53083, GSM340524, GSM80664, GSM175857, GSM80663, GSM175858, GSM80666, GSM175855, GSM80665, GSM175856, GSM80668, GSM175853, GSM179910, GSM80667, GSM175854, GSM176359, GSM199387, GSM317794, GSM316663, GSM176370, GSM176372, GSM176371, GSM351424, GSM175806, GSM350208, GSM175807, GSM175808, GSM175809, GSM179900, GSM175801, GSM389778, GSM175800, GSM175803, GSM122548, GSM152719, GSM175802, GSM175805, GSM53073, GSM175804, GSM176362, GSM176363, GSM203776, GSM176364, GSM345147, GSM176365, GSM199317, GSM176366, GSM176367, GSM306160, GSM176368, GSM176369, GSM176383, GSM176382, GSM176381, GSM316653, GSM350218, GSM351414, GSM95519, GSM389788, GSM95522, GSM95523, GSM95524, GSM53063, GSM95525, GSM152709, GSM176375, GSM199327, GSM176376, GSM95520, GSM345137, GSM176373, GSM203766, GSM95521, GSM176374, GSM176392, GSM345177, GSM170983, GSM176391, GSM170980, GSM176390, GSM95509, GSM95508, GSM350228, GSM175828, GSM175829, GSM95513, GSM80696, GSM175825, GSM95514, GSM80697, GSM53053, GSM175824, GSM170597, GSM199337, GSM95511, GSM80694, GSM175827, GSM170596, GSM122528, GSM95512, GSM80695, GSM175826, GSM170595, GSM95517, GSM175821, GSM95518, GSM175820, GSM95515, GSM80698, GSM175823, GSM95516, GSM80699, GSM175822, GSM306180, GSM170590, GSM176388, GSM176389, GSM80692, GSM170594, GSM176384, GSM95510, GSM80693, GSM170593, GSM176385, GSM80690, GSM170592, GSM176386, GSM80691, GSM170591, GSM176387, GSM203796, GSM170992, GSM345167, GSM350238, GSM175819, GSM53043, GSM53046, GSM175817, GSM175818, GSM95500, GSM175816, GSM95501, GSM175815, GSM95502, GSM175814, GSM199347, GSM95503, GSM175813, GSM95504, GSM175812, GSM170589, GSM95505, GSM175811, GSM170588, GSM95506, GSM175810, GSM95507, GSM306170, GSM345157, GSM203786, GSM176396, GSM385060, GSM73686, GSM76579, GSM345117, GSM337033, GSM158711, GSM385070, GSM345127, GSM76587, GSM76585, GSM340494, GSM96276, GSM337023, GSM76559, GSM361371, GSM60588, GSM176297, GSM176296, GSM337013, GSM361381, GSM158731, GSM114096, GSM76569, GSM335834, GSM345107, GSM176287, GSM155701, GSM176294, GSM176295, GSM176292, GSM176293, GSM176290, GSM176291, GSM337003, GSM158721, GSM175890, GSM175892, GSM175891, GSM175894, GSM175893, GSM175896, GSM175895, GSM89091, GSM60562, GSM175898, GSM175897, GSM175899, GSM385020, GSM306210, GSM155711, GSM361351, GSM385010, GSM152769, GSM390943, GSM270789, GSM337073, GSM89081, GSM155721, GSM361361, GSM385030, GSM306220, GSM387979, GSM152779, GSM337063, GSM175872,

GSM76595, GSM175871, GSM89071, GSM175874, GSM89072, GSM175873, GSM60548, GSM175870, GSM101100, GSM175879, GSM101101, GSM385040, GSM101102, GSM101103, GSM175876, GSM101104, GSM389824, GSM361331, GSM175875, GSM101105, GSM175878, GSM101106, GSM175877, GSM152789, GSM390158, GSM337053, GSM281029, GSM387969, GSM76590, GSM89060, GSM175885, GSM89061, GSM175884, GSM175883, GSM175882, GSM175881, GSM175880, GSM60538, GSM361341, GSM385050, GSM306200, GSM175889, GSM175888, GSM175887, GSM389813, GSM175886, GSM270799, GSM387959, GSM152799, GSM337043, GSM281039, GSM143900, GSM378170, GSM387949, GSM88971, GSM51690, GSM261312, GSM46948, GSM46941, GSM395790, GSM387939, GSM361321, GSM88981, GSM46938, GSM261302, GSM51680, GSM46936, GSM395780, GSM387929, GSM88991, GSM88997, GSM46928, GSM310839, GSM310838, GSM261332, GSM280009, GSM38103, GSM38104, GSM38100, GSM387919, GSM94603, GSM94604, GSM46918, GSM94605, GSM261322, GSM134589, GSM134588, GSM134587, GSM134586, GSM134584, GSM187595, GSM187596, GSM187593, GSM93568, GSM187594, GSM187599, GSM187597, GSM187598, GSM287293, GSM387909, GSM134591, GSM403597, GSM401092, GSM73656, GSM88949, GSM46975, GSM46976, GSM280028, GSM46973, GSM173691, GSM173690, GSM328997, GSM46960, GSM46961, GSM88955, GSM73666, GSM46968, GSM88951, GSM187586, GSM187587, GSM187588, GSM187589, GSM187584, GSM187585, GSM187590, GSM187592, GSM187591, GSM73676, GSM88961, GSM46958, GSM88962, GSM175903, GSM175904, GSM175901, GSM175902, GSM372348, GSM175900, GSM199417, GSM175909, GSM175908, GSM350308, GSM175907, GSM175906, GSM175905, GSM372358, GSM184639, GSM199427, GSM401062, GSM184636, GSM184637, GSM101095, GSM184638, GSM350318, GSM101096, GSM101097, GSM101098, GSM101099, GSM336033, GSM336983, GSM401076, GSM184640, GSM184641, GSM184644, GSM184645, GSM184642, GSM184643, GSM184648, GSM401072, GSM184649, GSM184646, GSM184647, GSM101998, GSM199407, GSM336043, GSM250001, GSM143898, GSM184650, GSM184651, GSM184652, GSM184653, GSM184654, GSM184655, GSM184656, GSM184657, GSM184658, GSM401082, GSM184659, GSM80900, GSM365142, GSM310849, GSM176409, GSM80901, GSM365143, GSM80902, GSM365140, GSM176407, GSM80903, GSM365141, GSM176408, GSM80904, GSM310845, GSM238951, GSM189790, GSM310846, GSM176406, GSM310847, GSM310848, GSM310844, GSM339558, GSM339559, GSM339566, GSM277701, GSM339565, GSM339568, GSM238949, GSM339567, GSM339562, GSM339561, GSM339564, GSM184665, GSM339563, GSM184664, GSM238943, GSM184663, GSM189782, GSM365139, GSM238944, GSM184662, GSM189783, GSM365138, GSM339560, GSM238941, GSM184661, GSM189784,

GSM365137, GSM238942, GSM184660, GSM189785, GSM365136, GSM238947, GSM189786, GSM365135, GSM238948, GSM189787, GSM365134, GSM238945, GSM189788, GSM365133, GSM238946, GSM189789, GSM80913, GSM365151, GSM336993, GSM176418, GSM365152, GSM176419, GSM80911, GSM365153, GSM80912, GSM365154, GSM310858, GSM176414, GSM189781, GSM310859, GSM176415, GSM189780, GSM176416, GSM365150, GSM310857, GSM176417, GSM176410, GSM176411, GSM310852, GSM176412, GSM310853, GSM176413, GSM46908, GSM310850, GSM310851, GSM339569, GSM387575, GSM189779, GSM277711, GSM365149, GSM189773, GSM365148, GSM189774, GSM189771, GSM189772, GSM365145, GSM189777, GSM365144, GSM189778, GSM365147, GSM189775, GSM365146, GSM189776, GSM365160, GSM176427, GSM365161, GSM176428, GSM176425, GSM189770, GSM176426, GSM365162, GSM176429, GSM387565, GSM310860, GSM176420, GSM310861, GSM310862, GSM176423, GSM176424, GSM176421, GSM176422, GSM189768, GSM189769, GSM365158, GSM189764, GSM365157, GSM189765, GSM365156, GSM189766, GSM365155, GSM189767, GSM189760, GSM189761, GSM238963, GSM189762, GSM365159, GSM189763, GSM176436, GSM176437, GSM176438, GSM176439, GSM176430, GSM176431, GSM94599, GSM176432, GSM94598, GSM176433, GSM176434, GSM176435, GSM339557, GSM189759, GSM189757, GSM189758, GSM189755, GSM189756, GSM189753, GSM189754, GSM238952, GSM189751, GSM238953, GSM189752, GSM238955, GSM187600, GSM345097, GSM125006, GSM187606, GSM187605, GSM187608, GSM187607, GSM187602, GSM187601, GSM187604, GSM187603, GSM242672, GSM175989, GSM242673, GSM158791, GSM176446, GSM100898, GSM175985, GSM150220, GSM176228, GSM176440, GSM187609, GSM176227, GSM242674, GSM175987, GSM150222, GSM76509, GSM242675, GSM175988, GSM169531, GSM150221, GSM176229, GSM176441, GSM175981, GSM150224, GSM176224, GSM175982, GSM150223, GSM176223, GSM175983, GSM150226, GSM176226, GSM175984, GSM150225, GSM176225, GSM176220, GSM176448, GSM150227, GSM176447, GSM176222, GSM175980, GSM176221, GSM176449, GSM345087, GSM176240, GSM176456, GSM175978, GSM176455, GSM175979, GSM176454, GSM175976, GSM176453, GSM175977, GSM176452, GSM175974, GSM176239, GSM176451, GSM175975, GSM176238, GSM176450, GSM176237, GSM175973, GSM176236, GSM176235, GSM176234, GSM176233, GSM176232, GSM100888, GSM176231, GSM176230, GSM391616, GSM365113, GSM365114, GSM125026, GSM365115, GSM365116, GSM365117, GSM365118, GSM345077, GSM365119, GSM277721, GSM176206, GSM176205, GSM175965, GSM176208, GSM363399, GSM175966, GSM176207, GSM363398, GSM175967,

GSM176466, GSM176209, GSM363396, GSM363395, GSM306240, GSM365121, GSM365120, GSM365124, GSM365125, GSM365122, GSM125016, GSM391626, GSM365123, GSM67153, GSM365128, GSM365129, GSM365126, GSM365127, GSM351339, GSM277731, GSM169530, GSM80567, GSM277094, GSM175954, GSM176219, GSM80566, GSM277095, GSM175955, GSM176218, GSM80569, GSM277092, GSM175952, GSM176217, GSM80568, GSM277093, GSM175953, GSM176216, GSM80563, GSM277098, GSM175958, GSM169525, GSM80562, GSM277099, GSM175959, GSM169524, GSM80565, GSM277096, GSM175956, GSM169527, GSM80564, GSM277097, GSM175957, GSM169526, GSM169529, GSM176211, GSM306230, GSM169528, GSM176210, GSM80561, GSM365132, GSM277090, GSM175950, GSM176215, GSM365131, GSM277091, GSM175951, GSM176214, GSM365130, GSM176213, GSM176212, GSM350348, GSM151324, GSM363383, GSM175949, GSM158741, GSM176271, GSM176270, GSM176273, GSM176272, GSM176267, GSM176268, GSM372301, GSM175940, GSM176269, GSM372300, GSM336013, GSM80571, GSM176263, GSM80572, GSM176264, GSM176265, GSM80570, GSM176266, GSM80575, GSM175946, GSM80576, GSM372306, GSM175945, GSM80573, GSM76549, GSM175948, GSM80574, GSM372308, GSM175947, GSM80579, GSM372303, GSM363379, GSM175942, GSM372302, GSM175941, GSM80577, GSM372305, GSM363377, GSM175944, GSM80578, GSM372304, GSM175943, GSM388709, GSM363390, GSM151314, GSM350358, GSM363392, GSM363394, GSM175938, GSM175939, GSM158751, GSM391606, GSM176280, GSM336023, GSM176278, GSM176279, GSM80580, GSM60601, GSM176276, GSM80581, GSM176277, GSM80582, GSM176274, GSM80583, GSM176275, GSM80584, GSM175937, GSM80585, GSM76539, GSM363385, GSM175936, GSM158761, GSM80586, GSM372318, GSM175935, GSM80587, GSM363387, GSM175934, GSM80588, GSM175933, GSM80589, GSM363389, GSM175932, GSM175931, GSM175930, GSM350328, GSM175927, GSM175928, GSM175929, GSM151344, GSM176251, GSM89101, GSM176250, GSM80593, GSM176241, GSM80594, GSM176242, GSM80591, GSM176243, GSM80592, GSM176244, GSM176245, GSM80590, GSM176246, GSM176247, GSM176248, GSM76529, GSM175920, GSM176249, GSM80599, GSM242653, GSM175922, GSM242652, GSM175921, GSM80597, GSM242651, GSM175924, GSM80598, GSM372328, GSM242650, GSM175923, GSM80595, GSM175926, GSM158771, GSM80596, GSM175925, GSM175918, GSM175919, GSM175916, GSM175917, GSM151334, GSM350338, GSM96266, GSM176262, GSM176261, GSM176260, GSM176254, GSM176255, GSM176252, GSM176253, GSM242668, GSM176258, GSM242667, GSM176259, GSM176256, GSM242669, GSM176257, GSM372338, GSM175911, GSM175910, GSM242666, GSM76519, GSM175915, GSM175914, GSM175913, GSM175912, GSM158781, GSM377475, GSM113822, GSM158811,

GSM85219, GSM85217, GSM85218, GSM371383, GSM85215, GSM85216, GSM199167, GSM350139, GSM125066, GSM148493, GSM113812, GSM148491, GSM148495, GSM148496, GSM158801, GSM357635, GSM371373, GSM199157, GSM125076, GSM148488, GSM335978, GSM148485, GSM125036, GSM148487, GSM199197, GSM350155, GSM350156, GSM199187, GSM350158, GSM102578, GSM350151, GSM350152, GSM350153, GSM350154, GSM125046, GSM335988, GSM159162, GSM371393, GSM350150, GSM350146, GSM102568, GSM350147, GSM199177, GSM350144, GSM350145, GSM350142, GSM249991, GSM350143, GSM350140, GSM350141, GSM350148, GSM125056, GSM350149, GSM277695, GSM158851, GSM277696, GSM114526, GSM176182, GSM176183, GSM176184, GSM114525, GSM176185, GSM176180, GSM176181, GSM176179, GSM51710, GSM176176, GSM176175, GSM176178, GSM176177, GSM249981, GSM151304, GSM158841, GSM114535, GSM176173, GSM176174, GSM176171, GSM176172, GSM261292, GSM176170, GSM387809, GSM114534, GSM261282, GSM176169, GSM51700, GSM176168, GSM176167, GSM176166, GSM176165, GSM176164, GSM277691, GSM249971, GSM113802, GSM114506, GSM158831, GSM114504, GSM114505, GSM125086, GSM261272, GSM387819, GSM249961, GSM85227, GSM85226, GSM85228, GSM158821, GSM85221, GSM85220, GSM85223, GSM85222, GSM85225, GSM114515, GSM85224, GSM114516, GSM125096, GSM176186, GSM387829, GSM261262, GSM249950, GSM402152, GSM335522, GSM150209, GSM386291, GSM249940, GSM312934, GSM161820, GSM102512, GSM80800, GSM287323, GSM261252, GSM387839, GSM361610, GSM102518, GSM371309, GSM371306, GSM371305, GSM371308, GSM371307, GSM371302, GSM327292, GSM371301, GSM371304, GSM371303, GSM249930, GSM150201, GSM150208, GSM161810, GSM335512, GSM161811, GSM287333, GSM161812, GSM161813, GSM361620, GSM312924, GSM102508, GSM387849, GSM102507, GSM261242, GSM327282, GSM150210, GSM161819, GSM249920, GSM161818, GSM161815, GSM161814, GSM161817, GSM161816, GSM312911, GSM312912, GSM155672, GSM312910, GSM155671, GSM287343, GSM387859, GSM261232, GSM312913, GSM312914, GSM361242, GSM161806, GSM161805, GSM161804, GSM161803, GSM249910, GSM161809, GSM155681, GSM161808, GSM161807, GSM312900, GSM312901, GSM287353, GSM312906, GSM312907, GSM312908, GSM387869, GSM312909, GSM261222, GSM312902, GSM312903, GSM312904, GSM312905, GSM155691, GSM249900, GSM183234, GSM261212, GSM387879, GSM102553, GSM102555, GSM102556, GSM155651, GSM102558, GSM183230, GSM386245, GSM335572, GSM387889, GSM155668, GSM155669, GSM261202, GSM155665, GSM155666, GSM155667, GSM183240, GSM102548, GSM155661, GSM155670,

GSM391596, GSM386255, GSM335562, GSM152009, GSM102538, GSM152006, GSM152005, GSM152008, GSM152007, GSM287303, GSM152002, GSM152001, GSM152004, GSM152003, GSM387899, GSM152000, GSM335552, GSM386225, GSM335938, GSM171597, GSM199027, GSM286700, GSM152017, GSM102528, GSM152016, GSM152015, GSM287313, GSM152014, GSM183220, GSM260703, GSM152013, GSM312944, GSM260702, GSM152012, GSM152011, GSM152010, GSM335532, GSM335542, GSM386235, GSM377465, GSM335942, GSM335941, GSM335940, GSM199037, GSM327202, GSM80868, GSM80867, GSM80869, GSM80874, GSM80870, GSM80871, GSM80872, GSM80873, GSM333446, GSM199047, GSM151294, GSM327212, GSM198042, GSM80887, GSM80888, GSM80885, GSM80886, GSM80883, GSM80884, GSM80881, GSM80882, GSM333436, GSM317934, GSM317933, GSM151284, GSM199057, GSM198052, GSM80845, GSM198053, GSM198050, GSM327222, GSM198051, GSM198049, GSM198048, GSM80851, GSM198047, GSM198046, GSM80853, GSM198045, GSM198044, GSM198043, GSM151274, GSM199067, GSM80861, GSM80865, GSM80866, GSM80864, GSM333456, GSM287383, GSM93939, GSM80823, GSM93938, GSM80824, GSM80825, GSM80826, GSM199077, GSM337202, GSM199087, GSM337203, GSM279998, GSM337200, GSM337201, GSM80831, GSM93944, GSM93943, GSM93941, GSM287373, GSM93946, GSM350413, GSM93948, GSM337205, GSM337204, GSM337207, GSM74882, GSM337206, GSM337209, GSM337208, GSM337210, GSM337211, GSM337212, GSM337213, GSM337214, GSM199097, GSM93954, GSM80844, GSM80843, GSM80842, GSM80841, GSM93950, GSM287363, GSM93952, GSM80801, GSM80802, GSM80803, GSM80804, GSM350423, GSM80805, GSM80806, GSM80807, GSM80808, GSM80809, GSM337219, GSM337218, GSM337217, GSM337216, GSM337215, GSM337224, GSM337225, GSM337222, GSM337223, GSM337220, GSM337221, GSM80811, GSM286660, GSM80810, GSM80814, GSM80815, GSM80812, GSM93927, GSM80813, GSM80818, GSM287393, GSM80819, GSM80816, GSM80817, GSM337227, GSM371403, GSM337226, GSM350433, GSM337229, GSM337228, GSM337233, GSM337234, GSM337235, GSM337236, GSM337230, GSM337231, GSM337232, GSM80822, GSM80821, GSM80820, GSM286650, GSM176128, GSM176129, GSM38094, GSM158891, GSM337241, GSM176120, GSM337240, GSM176121, GSM337243, GSM176122, GSM337242, GSM176123, GSM337245, GSM176124, GSM337244, GSM176125, GSM76640, GSM337247, GSM272315, GSM176126, GSM337246, GSM176127, GSM337237, GSM337238, GSM350443, GSM337239, GSM176130, GSM125106, GSM286690, GSM286670, GSM176139, GSM337250, GSM75563, GSM337254, GSM176133, GSM337253, GSM176134, GSM337252, GSM176131, GSM337251, GSM176132, GSM378160, GSM337258, GSM176137, GSM76630, GSM337257, GSM176138, GSM337256, GSM176135, GSM337255, GSM176136,

GSM337248, GSM48672, GSM350453, GSM337249, GSM176141, GSM176140, GSM286680, GSM337260, GSM158871, GSM75553, GSM119369, GSM176146, GSM176147, GSM337269, GSM176148, GSM176149, GSM176142, GSM89001, GSM176143, GSM176144, GSM176145, GSM176150, GSM74892, GSM242033, GSM176152, GSM242032, GSM176151, GSM350463, GSM337259, GSM158861, GSM277681, GSM158881, GSM119379, GSM176159, GSM337279, GSM176157, GSM176158, GSM176155, GSM199107, GSM176156, GSM89011, GSM176153, GSM176154, GSM176163, GSM350473, GSM176162, GSM176161, GSM176160, GSM175998, GSM175999, GSM175996, GSM175994, GSM277678, GSM175995, GSM175992, GSM175993, GSM175990, GSM175991, GSM38054, GSM89021, GSM76600, GSM179780, GSM337289, GSM350168, GSM359509, GSM199117, GSM50703, GSM139018, GSM139017, GSM139019, GSM151264, GSM179790, GSM89031, GSM242031, GSM38064, GSM337299, GSM38068, GSM350178, GSM119359, GSM119354, GSM199127, GSM179784, GSM179786, GSM89041, GSM139002, GSM176103, GSM139003, GSM176102, GSM139004, GSM176105, GSM139005, GSM176104, GSM80891, GSM80890, GSM76620, GSM176101, GSM176100, GSM38074, GSM199137, GSM80899, GSM176107, GSM80898, GSM350188, GSM176106, GSM80897, GSM176109, GSM176108, GSM80889, GSM103559, GSM89046, GSM150196, GSM150197, GSM150198, GSM150199, GSM139015, GSM176116, GSM139016, GSM176115, GSM139013, GSM176114, GSM89051, GSM139014, GSM176113, GSM139011, GSM176112, GSM139012, GSM176111, GSM76610, GSM176110, GSM139010, GSM350198, GSM38084, GSM199147, GSM176119, GSM176118, GSM176117, GSM139009, GSM139008, GSM139007, GSM125116, GSM139006, GSM194087, GSM194088, GSM194089, GSM203643, GSM194083, GSM194084, GSM96897, GSM194085, GSM203646, GSM96898, GSM158911, GSM194086, GSM343815, GSM159051, GSM187752, GSM281300, GSM231907, GSM231906, GSM194091, GSM194090, GSM102458, GSM194093, GSM194092, GSM102455, GSM387029, GSM312875, GSM102450, GSM102451, GSM203656, GSM158901, GSM194096, GSM194097, GSM194094, GSM194095, GSM261192, GSM343825, GSM231916, GSM159041, GSM187762, GSM261184, GSM249890, GSM281310, GSM102447, GSM199297, GSM102449, GSM102448, GSM387019, GSM312862, GSM158931, GSM203666, GSM159071, GSM211450, GSM158463, GSM158464, GSM187732, GSM377358, GSM231926, GSM349749, GSM211449, GSM249880, GSM387009, GSM176098, GSM176099, GSM312894, GSM102478, GSM312896, GSM312897, GSM312898, GSM312899, GSM211446, GSM281320, GSM211447, GSM199287, GSM211448, GSM194075, GSM158921, GSM159061, GSM194078, GSM194079, GSM203676, GSM402247, GSM194076, GSM194077, GSM176097, GSM187742,

GSM176096, GSM176095, GSM343805, GSM176094, GSM176093, GSM176092, GSM231936, GSM176091, GSM349739, GSM176090, GSM249870, GSM176089, GSM176087, GSM318094, GSM176088, GSM402257, GSM194082, GSM281330, GSM102468, GSM194081, GSM194080, GSM199277, GSM170833, GSM187792, GSM176080, GSM176081, GSM176082, GSM231946, GSM176083, GSM176084, GSM176085, GSM176086, GSM159091, GSM158951, GSM152569, GSM402267, GSM102498, GSM272305, GSM249860, GSM176077, GSM318084, GSM176076, GSM176079, GSM176078, GSM261151, GSM261152, GSM85506, GSM170835, GSM176070, GSM176071, GSM176074, GSM176075, GSM176072, GSM231956, GSM176073, GSM231950, GSM388192, GSM158941, GSM231952, GSM159081, GSM152579, GSM102488, GSM402277, GSM176068, GSM85513, GSM261146, GSM176067, GSM85514, GSM261143, GSM176066, GSM85515, GSM249850, GSM176065, GSM85516, GSM318074, GSM170823, GSM85517, GSM261142, GSM85518, GSM85519, GSM176069, GSM176061, GSM170850, GSM176062, GSM231966, GSM176063, GSM359583, GSM176064, GSM170855, GSM353428, GSM261182, GSM170853, GSM187772, GSM343837, GSM176060, GSM203626, GSM152589, GSM158971, GSM388182, GSM402287, GSM158981, GSM335602, GSM261172, GSM170858, GSM176059, GSM176058, GSM261174, GSM170857, GSM176055, GSM176054, GSM249840, GSM176057, GSM176056, GSM176052, GSM231976, GSM176053, GSM359593, GSM176050, GSM249820, GSM152594, GSM176051, GSM343847, GSM170841, GSM187782, GSM170844, GSM170843, GSM152599, GSM203636, GSM158961, GSM203641, GSM323169, GSM402297, GSM323168, GSM176049, GSM176048, GSM261162, GSM170848, GSM176047, GSM171011, GSM170849, GSM176046, GSM249830, GSM171012, GSM176045, GSM176044, GSM176043, GSM261113, GSM211032, GSM261112, GSM329007, GSM261117, GSM261116, GSM137954, GSM287463, GSM387731, GSM386393, GSM335622, GSM155968, GSM367219, GSM155969, GSM315621, GSM280907, GSM231986, GSM249810, GSM211042, GSM261102, GSM315622, GSM183301, GSM315623, GSM183300, GSM315624, GSM315625, GSM183302, GSM329017, GSM137964, GSM387741, GSM117629, GSM261109, GSM335612, GSM117632, GSM249800, GSM312816, GSM277128, GSM277129, GSM277126, GSM277127, GSM277125, GSM261134, GSM211052, GSM261132, GSM287443, GSM335642, GSM261138, GSM261137, GSM137934, GSM137931, GSM38376, GSM155989, GSM335652, GSM155988, GSM277132, GSM277131, GSM277130, GSM280927, GSM277137, GSM277138, GSM277139, GSM211062, GSM277133, GSM261122, GSM277134, GSM277135, GSM277136, GSM387721, GSM137945, GSM335632, GSM137944, GSM287453, GSM261127, GSM117649, GSM38386, GSM373559, GSM280917,

GSM137994, GSM277109, GSM287423, GSM277108, GSM277103, GSM277102, GSM277101, GSM277100, GSM277107, GSM277106, GSM277105, GSM277104, GSM201302, GSM377338, GSM201301, GSM201300, GSM155920, GSM277110, GSM280947, GSM201304, GSM201303, GSM155923, GSM155922, GSM155921, GSM38356, GSM155928, GSM155927, GSM287433, GSM155919, GSM387789, GSM158465, GSM158466, GSM158467, GSM158468, GSM312826, GSM158469, GSM353885, GSM377348, GSM158471, GSM280937, GSM158470, GSM158473, GSM158472, GSM158475, GSM158474, GSM335662, GSM38366, GSM287403, GSM102438, GSM353895, GSM280967, GSM155948, GSM155947, GSM287413, GSM137984, GSM102428, GSM312849, GSM211022, GSM211012, GSM280957, GSM101301, GSM38346, GSM117610, GSM80725, GSM272192, GSM80724, GSM272193, GSM80727, GSM327342, GSM272190, GSM80726, GSM335582, GSM272191, GSM80729, GSM386311, GSM80728, GSM280979, GSM138034, GSM272295, GSM183260, GSM80730, GSM239824, GSM80731, GSM239825, GSM80732, GSM272185, GSM239826, GSM80733, GSM80734, GSM272183, GSM80738, GSM335592, GSM80737, GSM386301, GSM272180, GSM80736, GSM272181, GSM80735, GSM327352, GSM272182, GSM117587, GSM80739, GSM337309, GSM280989, GSM138044, GSM80740, GSM272177, GSM80741, GSM286730, GSM272176, GSM183250, GSM272172, GSM80742, GSM272175, GSM80743, GSM272174, GSM327322, GSM183290, GSM386331, GSM272170, GSM53113, GSM272171, GSM80749, GSM80748, GSM280999, GSM138054, GSM272169, GSM134694, GSM272164, GSM272163, GSM272162, GSM272275, GSM272161, GSM286720, GSM272168, GSM80750, GSM80751, GSM272165, GSM386321, GSM183280, GSM80759, GSM327332, GSM80758, GSM53103, GSM80757, GSM272160, GSM134690, GSM134691, GSM134692, GSM134693, GSM272159, GSM134688, GSM272158, GSM134687, GSM134689, GSM272151, GSM272150, GSM272152, GSM272155, GSM272154, GSM183270, GSM272285, GSM272157, GSM80761, GSM387799, GSM286710, GSM272156, GSM337339, GSM201279, GSM401293, GSM201278, GSM201277, GSM316703, GSM53133, GSM137924, GSM201286, GSM201287, GSM201284, GSM201285, GSM201282, GSM201283, GSM201280, GSM201281, GSM119685, GSM119684, GSM119683, GSM119682, GSM179801, GSM201267, GSM119688, GSM179800, GSM201266, GSM119687, GSM201269, GSM337349, GSM119686, GSM201268, GSM119681, GSM53123, GSM119680, GSM316713, GSM137912, GSM137910, GSM80701, GSM80700, GSM138004, GSM201273, GSM138003, GSM201274, GSM119679, GSM138002, GSM201275, GSM201276, GSM137916, GSM201270, GSM137914, GSM201271, GSM201272, GSM179810, GSM201299, GSM337319, GSM80706, GSM53153, GSM117577, GSM80707, GSM80708, GSM316723, GSM80709, GSM80702, GSM80703, GSM80704, GSM80705, GSM80710, GSM80712, GSM80711,

GSM347925, GSM347924, GSM137904, GSM347923, GSM347922, GSM347921, GSM138014, GSM201289, GSM201288, GSM124996, GSM179820, GSM337329, GSM80719, GSM80717, GSM80718, GSM53143, GSM80715, GSM352629, GSM179827, GSM80716, GSM80713, GSM80714, GSM80723, GSM272194, GSM80722, GSM272195, GSM80721, GSM272196, GSM80720, GSM272197, GSM347916, GSM272198, GSM272199, GSM347918, GSM347917, GSM162960, GSM201290, GSM162961, GSM201291, GSM162962, GSM201292, GSM201293, GSM201294, GSM201295, GSM201296, GSM138024, GSM201297, GSM201298, GSM119649, GSM176025, GSM162954, GSM119648, GSM176026, GSM359603, GSM162957, GSM119647, GSM176027, GSM272215, GSM170867, GSM162956, GSM119646, GSM176028, GSM176021, GSM176022, GSM176023, GSM199217, GSM176024, GSM53173, GSM158991, GSM176029, GSM53170, GSM378838, GSM378837, GSM378836, GSM378831, GSM119651, GSM378830, GSM170862, GSM119652, GSM179830, GSM176031, GSM119650, GSM176030, GSM378835, GSM170865, GSM162958, GSM119655, GSM378834, GSM170866, GSM162959, GSM119656, GSM378833, GSM119653, GSM378832, GSM119654, GSM119636, GSM176038, GSM119635, GSM176039, GSM272225, GSM119638, GSM176036, GSM162943, GSM119637, GSM176037, GSM162942, GSM176034, GSM162941, GSM119639, GSM176035, GSM162940, GSM176032, GSM176033, GSM53163, GSM199227, GSM378826, GSM378825, GSM95473, GSM378828, GSM378827, GSM95475, GSM95474, GSM378829, GSM95477, GSM53167, GSM95476, GSM95479, GSM370399, GSM176042, GSM95478, GSM176041, GSM378820, GSM119640, GSM176040, GSM179840, GSM119641, GSM378822, GSM119642, GSM378821, GSM119643, GSM378824, GSM119644, GSM378823, GSM119645, GSM176000, GSM176001, GSM162931, GSM176002, GSM162930, GSM176003, GSM162933, GSM176004, GSM162932, GSM176005, GSM162935, GSM119669, GSM176006, GSM162934, GSM119668, GSM176007, GSM95480, GSM176008, GSM176009, GSM95488, GSM95487, GSM119670, GSM95486, GSM378819, GSM95485, GSM378818, GSM95484, GSM378817, GSM95483, GSM378816, GSM95482, GSM378815, GSM95481, GSM378814, GSM378813, GSM162936, GSM119677, GSM378812, GSM337359, GSM162937, GSM119678, GSM378811, GSM162938, GSM119675, GSM162939, GSM159101, GSM119673, GSM119674, GSM119671, GSM95489, GSM119672, GSM179850, GSM176012, GSM176013, GSM199207, GSM176010, GSM179870, GSM176011, GSM272205, GSM119658, GSM176016, GSM272204, GSM119657, GSM176017, GSM176014, GSM272202, GSM119659, GSM176015, GSM272201, GSM95490, GSM176018, GSM95491, GSM176019, GSM53183, GSM281280, GSM95497, GSM95496, GSM281290, GSM95499, GSM95498, GSM95493, GSM95492, GSM95495, GSM45796, GSM95494, GSM119664, GSM162928, GSM119665,

GSM337369, GSM159111, GSM119666, GSM119667, GSM119660, GSM176020, GSM179860, GSM119661, GSM162929, GSM119662, GSM119663, GSM272143, GSM301693, GSM272144, GSM272145, GSM152619, GSM80771, GSM272146, GSM199257, GSM80778, GSM80777, GSM272140, GSM80776, GSM272255, GSM272141, GSM272142, GSM272147, GSM179880, GSM272148, GSM272149, GSM159122, GSM327302, GSM301687, GSM80783, GSM272134, GSM80782, GSM272135, GSM80785, GSM80784, GSM152609, GSM80787, GSM80786, GSM301680, GSM80789, GSM199267, GSM80788, GSM350078, GSM272265, GSM162902, GSM272138, GSM272139, GSM179890, GSM80781, GSM272136, GSM80780, GSM272137, GSM162906, GSM162905, GSM162904, GSM159132, GSM399579, GSM80779, GSM327312, GSM301677, GSM80799, GSM80798, GSM80797, GSM80796, GSM80795, GSM199237, GSM80794, GSM80793, GSM80792, GSM80791, GSM80790, GSM119628, GSM119629, GSM272235, GSM249790, GSM119626, GSM119627, GSM119624, GSM119625, GSM119634, GSM119633, GSM119632, GSM119631, GSM119630, GSM159142, GSM152639, GSM238763, GSM301667, GSM272245, GSM199247, GSM152629, GSM119617, GSM119618, GSM119619, GSM119615, GSM119616, GSM119621, GSM119620, GSM119623, GSM119622, GSM159152, GSM301657, GSM152624, GSM97793, GSM97794, GSM97795, GSM97796, GSM97797, GSM97798, GSM97799, GSM97800, GSM97801, GSM97802, GSM97803, GSM97804, GSM97805, GSM97806, GSM97807, GSM97808, GSM97809, GSM97810, GSM97811, GSM97812, GSM97813, GSM97814, GSM97815, GSM97816, GSM97817, GSM97818, GSM97819, GSM97820, GSM97821, GSM97822, GSM97823, GSM97824, GSM97825, GSM97826, GSM97827, GSM97828, GSM97829, GSM97830, GSM97831, GSM97832, GSM97833, GSM97834, GSM97835, GSM97836, GSM97837, GSM97838, GSM97839, GSM97840, GSM97841, GSM97842, GSM97843, GSM97844, GSM97845, GSM97846, GSM97847, GSM97848, GSM97849, GSM97850, GSM97851, GSM97852, GSM97853, GSM97854, GSM97855, GSM97856, GSM97857, GSM97858, GSM97859, GSM97860, GSM97861, GSM97862, GSM97863, GSM97864, GSM97865, GSM97866, GSM97867, GSM97868, GSM97869, GSM97870, GSM97871, GSM97872, GSM97873, GSM97874, GSM97875, GSM97876, GSM97877, GSM97878, GSM97879, GSM97880, GSM97881, GSM97882, GSM97883, GSM97884, GSM97885, GSM97886, GSM97887, GSM97888, GSM97889, GSM97890, GSM97891, GSM97892, GSM97893, GSM97894, GSM97895, GSM97896, GSM97897, GSM97898, GSM97899, GSM97900, GSM97901, GSM97902, GSM97903, GSM97904, GSM97905, GSM97906, GSM97907, GSM97908, GSM97909, GSM97910, GSM97911, GSM97912, GSM97913, GSM97914, GSM97915, GSM97916, GSM97917, GSM97918, GSM97919, GSM97920, GSM97921, GSM97922, GSM97923, GSM97924, GSM97925, GSM97926, GSM97927, GSM97928, GSM97929, GSM97930, GSM97931, GSM97932, GSM97933, GSM97934, GSM97935, GSM97936, GSM97937, GSM97938,

GSM97939, GSM97940, GSM97941, GSM97942, GSM97943, GSM97944, GSM97945, GSM97946, GSM97947, GSM97948, GSM97949, GSM97950, GSM97951, GSM97952, GSM97953, GSM97954, GSM97955, GSM97956, GSM97957, GSM97958, GSM97959, GSM97960, GSM97961, GSM97962, GSM97963, GSM97964, GSM97965, GSM97966, GSM97967, GSM97968, GSM97969, GSM97970, GSM97971, GSM97972
